# *De novo* variants in *DENND5B* cause a neurodevelopmental disorder

**DOI:** 10.1016/j.ajhg.2024.02.001

**Published:** 2024-02-21

**Authors:** Marcello Scala, Valeria Tomati, Matteo Ferla, Mariateresa Lena, Julie S. Cohen, Ali Fatemi, Elly Brokamp, Anna Bican, John A. Phillips, Mary E. Koziura, Michael Nicouleau, Marlene Rio, Karine Siquier, Nathalie Boddaert, Ilaria Musante, Serena Tamburro, Simona Baldassari, Michele Iacomino, Paolo Scudieri, Undiagnosed Diseases Network, Maria T. Acosta, David R. Adams, Raquel L. Alvarez, Justin Alvey, Aimee Allworth, Ashley Andrews, Euan A. Ashley, Ben Afzali, Carlos A. Bacino, Guney Bademci, Ashok Balasubramanyam, Dustin Baldridge, Jim Bale, Michael Bamshad, Deborah Barbouth, Pinar Bayrak-Toydemir, Anita Beck, Alan H. Beggs, Edward Behrens, Gill Bejerano, Hugo J. Bellen, Jimmy Bennett, Jonathan A. Bernstein, Gerard T. Berry, Anna Bican, Stephanie Bivona, Elizabeth Blue, John Bohnsack, Devon Bonner, Lorenzo Botto, Lauren C. Briere, Gabrielle Brown, Elizabeth A. Burke, Lindsay C. Burrage, Manish J. Butte, Peter Byers, William E. Byrd, John Carey, Olveen Carrasquillo, Thomas Cassini, Ta Chen Peter Chang, Sirisak Chanprasert, HsiaoTuan Chao, Ivan Chinn, Gary D. Clark, Terra R. Coakley, Laurel A. Cobban, Joy D. Cogan, Matthew Coggins, F. Sessions Cole, Heather A. Colley, Heidi Cope, Rosario Corona, William J. Craigen, Andrew B. Crouse, Michael Cunningham, Precilla D’Souza, Hongzheng Dai, Surendra Dasari, Joie Davis, Jyoti G. Dayal, Margaret Delgado, Esteban C. Dell'Angelica, Katrina Dipple, Daniel Doherty, Naghmeh Dorrani, Argenia L. Doss, Emilie D. Douine, Dawn Earl, David J. Eckstein, Lisa T. Emrick, Christine M. Eng, Marni Falk, Elizabeth L. Fieg, Paul G. Fisher, Brent L. Fogel, Irman Forghani, Jiayu Fu, William A. Gahl, Ian Glass, Page C. Goddard, Rena A. Godfrey, Alana Grajewski, Andrea Gropman, Meghan C. Halley, Rizwan Hamid, Neal Hanchard, Kelly Hassey, Nichole Hayes, Frances High, Anne Hing, Fuki M. Hisama, Ingrid A. Holm, Jason Hom, Martha Horike-Pyne, Alden Huang, Yan Huang, Sarah Hutchison, Wendy Introne, Rosario Isasi, Kosuke Izumi, Gail P. Jarvik, Jeffrey Jarvik, Suman Jayadev, Orpa Jean-Marie, Vaidehi Jobanputra, Emerald Kaitryn, Shamika Ketkar, Dana Kiley, Gonench Kilich, Shilpa N. Kobren, Isaac S. Kohane, Jennefer N. Kohler, Susan Korrick, Deborah Krakow, Donna M. Krasnewich, Elijah Kravets, Seema R. Lalani, Byron Lam, Christina Lam, Brendan C. Lanpher, Ian R. Lanza, Kimberly LeBlanc, Brendan H. Lee, Roy Levitt, Richard A. Lewis, Pengfei Liu, Xue Zhong Liu, Nicola Longo, Sandra K. Loo, Joseph Loscalzo, Richard L. Maas, Ellen F. Macnamara, Calum A. MacRae, Valerie V. Maduro, AudreyStephannie Maghiro, Rachel Mahoney, May Christine V. Malicdan, Laura A. Mamounas, Teri A. Manolio, Rong Mao, Ronit Marom, Gabor Marth, Beth A. Martin, Martin G. Martin, Julian A. Martínez-Agosto, Shruti Marwaha, Jacob McCauley, Allyn McConkie-Rosell, Alexa T. McCray, Elisabeth McGee, Matthew Might, Danny Miller, Ghayda Mirzaa, Eva Morava, Paolo Moretti, Marie Morimoto, John J. Mulvihill, Mariko Nakano-Okuno, Stanley F. Nelson, Shirley Nieves-Rodriguez, Donna Novacic, Devin Oglesbee, James P. Orengo, Laura Pace, Stephen Pak, J. Carl Pallais, Jeanette C. Papp, Neil H. Parker, Leoyklang Petcharet, John A. Phillips, Jennifer E. Posey, Lorraine Potocki, Barbara N. Pusey Swerdzewski, Aaron Quinlan, Deepak A. Rao, Anna Raper, Wendy Raskind, Genecee Renteria, Chloe M. Reuter, Lynette Rives, Amy K. Robertson, Lance H. Rodan, Jill A. Rosenfeld, Elizabeth Rosenthal, Francis Rossignol, Maura Ruzhnikov, Marla Sabaii, Ralph Sacco, Jacinda B. Sampson, Mario Saporta, Judy Schaechter, Timothy Schedl, Kelly Schoch, Daryl A. Scott, Elaine Seto, Prashant Sharma, Vandana Shashi, Emily Shelkowitz, Sam Sheppeard, Jimann Shin, Edwin K. Silverman, Janet S. Sinsheimer, Kathy Sisco, Edward C. Smith, Kevin S. Smith, Lilianna Solnica-Krezel, Ben Solomon, Rebecca C. Spillmann, Andrew Stergachis, Joan M. Stoler, Kathleen Sullivan, Jennifer A. Sullivan, Shirley Sutton, David A. Sweetser, Virginia Sybert, Holly K. Tabor, Queenie K.-G. Tan, Amelia L.M. Tan, Arjun Tarakad, Herman Taylor, Mustafa Tekin, Fred Telischi, Willa Thorson, Cynthia J. Tifft, Camilo Toro, Alyssa A. Tran, Rachel A. Ungar, Tiina K. Urv, Adeline Vanderver, Matt Velinder, Dave Viskochil, Tiphanie P. Vogel, Colleen E. Wahl, Melissa Walker, Nicole M. Walley, Jennifer Wambach, Jijun Wan, Lee-kai Wang, Michael F. Wangler, Patricia A. Ward, Daniel Wegner, Monika Weisz Hubshman, Mark Wener, Tara Wenger, Monte Westerfield, Matthew T. Wheeler, Jordan Whitlock, Lynne A. Wolfe, Kim Worley, Shinya Yamamoto, Zhe Zhang, Stephan Zuchner, Jill A. Rosenfeld, Gary Bellus, Sara Reed, Hind Al Saif, Rossana Sanchez Russo, Matthew B. Walsh, Vincent Cantagrel, Amy Crunk, Stefano Gustincich, Sarah M. Ruggiero, Mark P. Fitzgerald, Ingo Helbig, Pasquale Striano, Mariasavina Severino, Vincenzo Salpietro, Nicoletta Pedemonte, Federico Zara

**Affiliations:** 1 Department of Neurosciences, Rehabilitation, Ophthalmology, Genetics, Maternal and Child Health, University of Genoa, Genoa, Italy; 2 Pediatric Neurology and Muscular Diseases Unit, IRCCS Istituto Giannina Gaslini, Genoa, Italy; 3 UOC Genetica Medica, IRCCS Giannina Gaslini, Genoa, Italy; 4 Oxford Protein Informatics Group, Department of Statistics, University of Oxford, Oxford, UK; 5 Department of Neurology and Developmental Medicine, Kennedy Krieger Institute, Baltimore, MD, USA; 6 Department of Neurology, Johns Hopkins University School of Medicine, Baltimore, MD, USA; 7 Department of Pediatrics, Vanderbilt University Medical Center, Nashville, TN, USA; 8 Université Paris Cité, Imagine Institute, Developmental Brain Disorders Laboratory, INSERM UMR 1163, 75015 Paris, France; 9 Service de Génétique, Necker Enfants Malades University Hospital, Assistance Publique-Hôpitaux de Pairs, Paris, France; 10 Département de Radiologie Pédiatrique, INSERM UMR 1163 and INSERM U1000, AP-HP, Hôpital Necker-Enfants Malades, Paris, France; 11 Department of Molecular & Human Genetics, Baylor College of Medicine, Houston, TX, USA; 12 Baylor Genetics Laboratories, Houston, TX, USA; 13 Clinical Genetics, Geisinger Medical Center, Danville, PA 17822, USA; 14 Department of Human and Molecular Genetics, Division of Clinical Genetics, Virginia Commonwealth University School of Medicine, Richmond, VA, USA; 15 Department of Human Genetics, Emory University, Atlanta, GA 30322, USA; 16 GeneDx, Gaithersburg, MD 20877, USA; 17 Department of Neuroscience and Brain Technologies, Istituto Italiano di Tecnologia, 16163 Genoa, Italy; 18 Division of Neurology, Children’s Hospital of Philadelphia, Philadelphia, PA 19104, USA; 19 The Epilepsy NeuroGenetics Initiative (ENGIN), Children’s Hospital of Philadelphia, Philadelphia, PA, USA; 20 Department of Neurology, University of Pennsylvania, Perelman School of Medicine, Philadelphia, PA 19104, USA; 21 Department of Biomedical and Health Informatics (DBHi), Children’s Hospital of Philadelphia, Philadelphia, PA 19104, USA; 22 Neuroradiology Unit, IRCCS Istituto Giannina Gaslini, Genoa, Italy

**Keywords:** DENND5B, guanine nucleotide exchange factors, Rab GPTases, cell homeostasis, neurodevelopmental disorder, intellectual disability, epilepsy, membrane trafficking, lipid uptake and distribution

## Abstract

The Rab family of guanosine triphosphatases (GTPases) includes key regulators of intracellular transport and membrane trafficking targeting specific steps in exocytic, endocytic, and recycling pathways. DENND5B (Rab6-interacting Protein 1B-like protein, R6IP1B) is the longest isoform of DENND5, an evolutionarily conserved DENN domain-containing guanine nucleotide exchange factor (GEF) that is highly expressed in the brain. Through exome sequencing and international matchmaking platforms, we identified five *de novo* variants in *DENND5B* in a cohort of five unrelated individuals with neurodevelopmental phenotypes featuring cognitive impairment, dysmorphism, abnormal behavior, variable epilepsy, white matter abnormalities, and cortical gyration defects. We used biochemical assays and confocal microscopy to assess the impact of DENND5B variants on protein accumulation and distribution. Then, exploiting fluorescent lipid cargoes coupled to high-content imaging and analysis in living cells, we investigated whether DENND5B variants affected the dynamics of vesicle-mediated intracellular transport of specific cargoes. We further generated an *in silico* model to investigate the consequences of DENND5B variants on the DENND5B-RAB39A interaction. Biochemical analysis showed decreased protein levels of DENND5B mutants in various cell types. Functional investigation of DENND5B variants revealed defective intracellular vesicle trafficking, with significant impairment of lipid uptake and distribution. Although none of the variants affected the DENND5B-RAB39A interface, all were predicted to disrupt protein folding. Overall, our findings indicate that *DENND5B* variants perturb intracellular membrane trafficking pathways and cause a complex neurodevelopmental syndrome with variable epilepsy and white matter involvement.

## Graphical abstract

**Figure:**
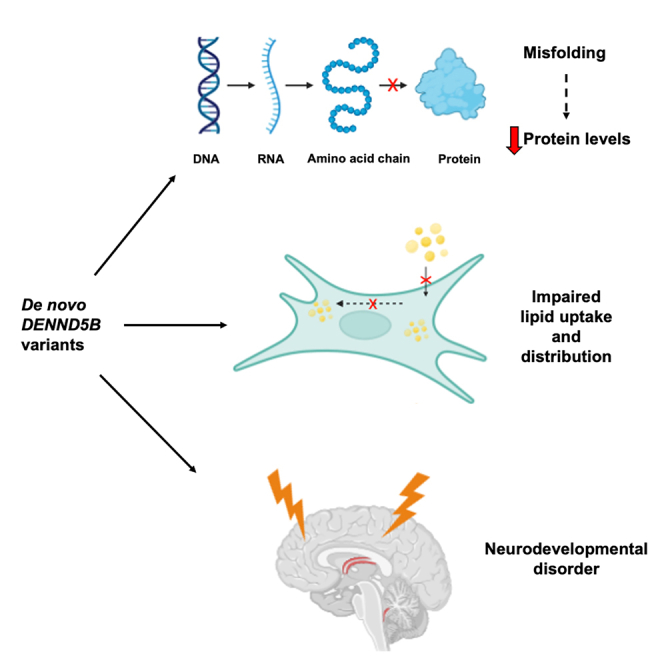

## Teaser

In this study, we identify *de novo* missense variants in *DENND5B* as the cause of a neurodevelopmental disorder with dysmorphism and epilepsy. We show that these variants impair intracellular vesicle trafficking, lipid uptake and intracellular distribution, and protein folding, leading to complex neurological manifestations.

## Introduction

The differentially expressed in normal and neoplastic (DENN) cells domain is a poorly characterized though extremely conserved module present in proteins of diverse species.^1^ In the human genome, eighteen genes encode DENN domain-containing proteins that function as generalized guanine nucleotide exchange factors (GEFs) for Rab family proteins.^2^ These are small guanosine triphosphatases (GTPases) regulating membrane trafficking through the orchestration of biogenesis, transport, and fusion of intracellular organelles and vesicles.^3^^,^^4^ Acting as molecular switches, Rab proteins cycle between an active GTP-loaded state and an inactive GDP-loaded state, with ancillary GEFs and GTPase-activating proteins (GAPs) catalyzing this cycle.^3^^,^^4^ As orchestrators of intracellular vesicle movement, Rab proteins are essential regulators of neuronal polarity and modulators of synaptic architecture and function.^5^ The functional deficiency of Rab proteins is associated with heterogeneous human diseases, including neurodevelopmental disorders (NDDs) and neurodegenerative conditions.^6^

*DENND5B* (MIM: 617279) is located on chromosome 12 (12p11.21) and encodes a 1,274-amino-acid protein with a molecular weight of 145 kDa.^7^^,^^8^ DENND5B, also known as Rab6-interacting Protein 1B-like protein (R6IP1B or R6IP1-like), is one of the two members of the DENND5 protein subfamily, the other being DENND5A (also known as R6IP1A or R6IP1).^7^^,^^8^ These related DENN proteins interact with functionally relevant Rab proteins, such as RAB6, RAB11, and RAB39. As such, DENND5 proteins have been suggested to contribute to synaptic plasticity through the modulation of synaptic vesicle axonal trafficking and neurotransmitter release.^7^^,^^8^ DENND5A and DENND5B contain six distinct functional domains. The DENN domain is encircled on both sides by more divergent domains, known as uDENN (upstream DENN) and dDENN (downstream DENN), forming the tripartite DENN domain. This evolutionarily conserved module mainly exerts the GEF activity toward Rab proteins, with the uDENN module also responsible for the interaction between DENND5A and RAB11.^1^^,^^2^^,^^8^ The first of the two RUN (RPIP8 [RaP2-interacting protein 8], UNC-14, and NESCA [new molecule containing SH3 at the carboxyl terminus]) domains, RUN1, mediates the nucleotide-dependent binding to Rab6, that is a crucial step for Rab6-dependent targeting of DENND5B to the Golgi.^1^^,^^8^ Based on the similarities with DENND5A, the RUN2 domain of DENND5B is predicted to interact with Sorting Nexin 1.^9^ SNX1 is a conserved membrane-associated protein that regulates the retrograde transport of a large number of cargoes from endosome to trans-Golgi network (TGN).^10^ Thus, it is possible that DENND5B contributes to regulate cargo trafficking in the endosome-to-TGN pathway.^9^ RUN1-2 domains are separated by a PLAT (polycystin-1, lipoxygenase, alpha-toxin) domain, whose lipophilic loops interacts with lipids within the membranes of recycling endosomes and may contribute to the tethering of these membranes with Golgi membranes together with RAB11.^11^

Recent evidence suggests that DENND5B may be implicated in the regulation of lipid metabolism. Studies focusing on the *Dennd5b*^*−/−*^ mouse revealed that DENND5B plays a crucial role in post-Golgi chylomicron secretion, contributing to modulate intestinal triglyceride absorption, body composition, and peripheral lipoprotein metabolism.^12^ Additionally, DENND5B deficiency leads to differential expression of key genes involved in hepatic lipid metabolism and lipid storage, resistance to diet-induced obesity, overall increase in plasma lipids, and reduced aortic atherosclerosis.^13^ In human subjects, the presence of the polymorphic variant (GenBank: NM_144973.4): c.155G>A (p.Arg52Lys) (rs4930979, AF = 0.441) in homozygous status has been associated with low body mass index (BMI) and reduced abdominal circumference.^12^^,^^14^^,^^15^ Another polymorphism, GenBank: NM_144973.4 (c.1459C>A [p.His487Asn]) (AF = 0.137), was instead correlated with low-density lipoprotein cholesterol (LDL-C) plasmatic profile but not to BMI.^12^^,^^13^

Aside from this influence on lipid metabolism, DENND5 is suggested to play a relevant role in brain development and function. *Dennd5a* knockdown in mouse hippocampal neurons resulted in dendrite outgrowth and improper synaptic connectivity.^16^ Humans harboring bi-allelic loss-of-function variants in *DENND5A* (MIM: 617278) show a severe epileptic encephalopathy with brain calcifications (developmental and epileptic encephalopathy 49 [MIM: 617281]).^16^ In mice, *Dennd5b* expression is mainly restricted to neurons and is especially enriched in the hippocampus.^7^ In the human brain, *DENND5B* is preferentially expressed in the cerebral cortex, basal ganglia, cerebellum, and hippocampus (https://www.proteinatlas.org/ENSG00000170456-DENND5B/tissue). Additionally, the expression of *DENND5B* was recently reported to be significantly decreased in the brains of individuals with temporal lobe epilepsy and chronic epileptic mouse models.^7^ Overall, these findings suggest that DENND5B function may correlate with epileptogenesis and, possibly, neurodevelopment in humans.^7^

In this study, we investigated a cohort of five unrelated individuals presenting with neurodevelopmental phenotypes featuring abnormal behavior, seizures, and distinct brain MRI abnormalities. In these subjects, we identified five *de novo* missense variants in *DENND5B*. Using different biochemical and functional approaches, we investigated the impact of selected *DENND5B* variants on protein accumulation, endosomal system activity, and intracellular membrane trafficking pathways. Our findings highlight the role of *DENND5B* variants as the cause of a neurodevelopmental syndrome with early-onset seizures and white matter abnormalities.

## Subjects and methods

### Ethics statement

The study involving human participants was conducted according to the guidelines of the Declaration of Helsinki and was approved by the medical ethical committee installed by Gaslini Children’s Hospital (Comitato Etico della Regione Liguria, approval code 163/2018). Informed consent was obtained from the parents or legal guardians of all the enrolled participants.

### Subject enrollment and ascertainment

Affected individuals were recruited through international collaboration involving several clinical and research centers in Italy, the Netherlands, and the USA (further details available in the supplemental information), also using match maker platforms such as GeneMatcher.^17^ Enrolled subjects were thoroughly evaluated by pediatric neurologists, neuropsychiatrists, and neurogeneticists. The degree of intellectual disability was evaluated through Wechsler Intelligence Scale for Children (WISC V) in subjects #1 and #2, and thorough neuropsychiatric evaluation was performed to assess the degree of neurodevelopmental impairment in the remaining individuals. Brain MRI scans, locally performed for care purposes, were reviewed by an expert pediatric neuroradiologist (M. Severino).

### Genetic analysis

For exome sequencing (ES), genomic DNA was extracted from peripheral blood lymphocytes or buccal swab samples of the probands and parents (when available). Trio-ES was performed in all subjects as previously described (supplemental information). Variants were filtered according to minor allele frequency ≤0.001 in Genome Aggregation Database (gnomAD v.4.0.0, https://gnomad.broadinstitute.org), conservation of affected residues (Genomic Evolutionary Rate Profiling [GERP], http://mendel.stanford.edu/SidowLab/downloads/gerp/), and predicted functional impact through different *in silico* tools. Candidate variants were classified according to the American College of Medical Genetics and Genomics and the Association for Molecular Pathology (ACMG-AMP) guidelines, based on their predicted pathogenicity.^18^ *DENND5B* variants are reported according to GenBank: NM_144973.4 transcript, corresponding to the isoform 1 of DENND5B protein (GenBank: NP_659410.3). Further details are available in the supplemental information. The presence of copy number variations (CNVs) was investigated in all subjects through array comparative genomic hybridization (aCGH) or ES-based CNV calling (supplemental information).

### Cell culture

HeLa cells were grown in DMEM (Euroclone) cell culture medium, while CFPAC-1 cells were grown in DMEM-F12 (Euroclone). Cell culture media were supplemented with 10% FBS (ECS5000L Euroclone), 2 mM L-glutamine, 100 U/mL penicillin, and 100 μg/mL streptomycin (Euroclone). Primary fibroblasts were grown in RPMI medium (Euroclone) supplemented with 20% FBS (ECS5000L Euroclone), 2 mM L-glutamine, 100 U/mL penicillin, and 100 μg/mL streptomycin (Euroclone). For sphingolipid transport functional analysis, for autophagic vacuoles detection, and for DENND5B immunolocalization studies, 30 × 10^5^ per well primary fibroblasts derived from affected individuals’ dermal biopsies, HeLa, or CFPAC-1 cells were plated in 96-wells plates (Corning). For CFPAC-1 cells, plates were coated with poly-D-lysine (Merck KGaA) to increase cellular adherence to the bottom of the wells. Cells were imaged 24 h after plating.

### Vectors

We have designed a custom reporter backbone containing a CMV promoter, *DENND5B* (encoding either wild-type or the variant forms p.Ser800Leu, p.Asp849Glu, p.His852Tyr) followed by an engineered Tag Blue Fluorescent Protein 2 (TagBFP2) BFP2 (an improved variant of TagBFP generated by I174A mutation) fused with the neomycin resistance gene *Neo* (TagBFP2/*Neo*) as expression marker.^19^ The generation of the plasmid was outsourced to VectorBuilder (vector IDs available upon request).

### Transient transfection

For sphingolipid transport functional analysis and for DENND5B immunolocalization studies, HeLa and CFPAC-1 cells were reverse-transfected onto 96-well plates (Corning) with 0.2 μg per well of the indicated vectors (see subjects and methods, vectors section). To measure DENND5B accumulation by western blot, HeLa and CFPAC-1 cells were reverse-transfected onto 6-well plates with 2 μg per well of the indicated vectors (see subjects and methods, vectors section). Lipofectamine 2000 (ThermoFisher Scientific) was used as a transfection reagent. Cells were transfected in Opti- MEM Reduced Serum Medium (ThermoFisher Scientific). After 6 h, Opti-MEM was carefully replaced with culture medium without antibiotics.

### Western blot

To measure DENND5B accumulation by western blot, primary fibroblast derived from affected individuals’ dermal biopsies (#2 and #3) were grown to confluence onto a 60 mm Petri dish, while HeLa and CFPAC-1 cells were grown to confluence onto a 6-well plate. The day of cell lysis, cells were washed with ice-cold D-PBS without Ca^2+^/Mg^2+^ and then lysed in RIPA buffer 50 mM (50 mM Tris-HCl [pH 7.4], 150 mM NaCl, 1% Triton X-100, 0.5% sodium deoxycholate, 0.1% SDS) containing a complete protease inhibitor cocktail (Roche). Lysates were then separated by centrifugation at 15,000 × g at 4°C for 10 min. The supernatant protein concentration was calculated using a BCA assay (ThermoFisher Scientific) following the manufacturer’s protocol. 30 μg of total cell lysates were separated onto gradient 4%–15% Criterion TGX Precast gels (Bio-rad Laboratories), transferred to a nitrocellulose membrane with a Trans-Blot Turbo system (Bio-rad Laboratories), and analyzed by western blotting. DENND5B and GAPDH were detected using the following antibodies: rabbit polyclonal anti-DENND5B (PA5-58569, Invitrogen, ThermoFisher Scientific), rabbit polyclonal anti-DENND5B (HPA038865, Merck KGaA), and mouse monoclonal anti-GAPDH (sc-32233; Santa Cruz Biotechnology; RRID: AB_627679). Membranes were subsequently incubated with secondary antibodies horseradish peroxidase (HRP)-conjugated anti-rabbit IgG (31460; ThermoFisher Scientific; RRID: AB_228341) or HRP-conjugated anti-mouse IgG (ab97023; Abcam; RRID: AB_10679675). Proteins were visualized by chemiluminescence using the SuperSignalWest Femto Substrate (ThermoFisher Scientific). Molecular Imager ChemiDoc XRS System (Bio-rad Laboratories) was used to monitor the chemiluminescence. Images were analyzed with ImageJ software (National Institutes of Health). Bands were analyzed as region of interest (ROI), normalized against the GAPDH loading control.

### Sphingolipid transport functional analysis

HeLa cells, transiently expressing the WT or *DENND5B* mutants, or fibroblasts derived from subjects with different genotypes, plated on clear-bottom 96-well black microplates suitable for high-content imaging, were exposed, at the beginning of the experiments, to fluorescent lipid probes (NBD C6-ceramide [catalog #N1154] or BODIPY FL C12-sphingomyelin [catalog #D7711], ThermoFisher Scientific) that were added to the medium. The uptake and intracellular distribution of the fluorescent probes was then monitored by high-content imaging and analysis using an Opera Phenix (PerkinElmer) high-content screening system. Wells were imaged in confocal mode, using a 40× water-immersion objective. NBD or BODIPY fluorescent signals were laser-excited at 480 nm and the emission wavelengths were collected between 500 and 550 nm. Fluorescent spots resembling intracellular vesicles containing fluorescent lipids were quantified using the Harmony software (v.4.9) of the Opera Phenix high-content system. For each DENND5B form, transiently transfected in HeLa cells, five independent replicates were analyzed. Each independent biological replicate consisted of a region measuring 1,320 × 1,320 μm, comprising approximately 35–40 TagBFP2-positive cells. For each patient-derived fibroblast population, nine independent replicates were analyzed. Each independent biological replicate consisted of a region measuring 990 × 1,320 μm, comprising approximately one hundred fibroblasts. Statistical significance was tested by parametric ANOVA followed by the Dunnet multiple comparisons test (all groups against the control group).

### Autophagic vacuoles detection

HeLa cells, transiently transfected with plasmids encoding WT or DENND5B mutants and plated on clear-bottom 96-well black microplates suitable for high-content imaging, were incubated with 50 μM MDC (Sigma-Aldrich) in PBS at 37°C for 10 min. After incubation, cells were washed three times with PBS and immediately imaged, in confocal mode and with a 40× water-immersion objective, using an Opera Phenix (PerkinElmer) high-content screening system. MDC signal was laser-excited at 405 nm and the emission wavelengths were collected between 435 and 550 nm. Data analysis of MDC spot number was performed using the Harmony software (v.4.9) of the Opera Phenix high-content system. For each DENND5B form, transiently transfected in HeLa cells, five independent replicates were analyzed. Each independent biological replicate consisted of a region measuring 1,320 × 1,320 μm, comprising approximately 35–40 TagBFP2-positive cells. Statistical significance was tested by parametric ANOVA followed by the Dunnet multiple comparisons test (all groups against the control group).

### Statistical analysis

Statistical significance was assessed by performing the analysis of variance (ANOVA) followed by a post hoc test, to avoid “multiple comparison errors” when comparing more than two groups. The assumption of normality was assessed using the Kolmogorov-Smirnov test. In case of normally distributed quantitative variables, a parametric ANOVA was performed. Data derived from biochemical and functional analyses on heterologous expression systems or fibroblasts derived from affected individuals, normally distributed, are expressed as mean ± SEM. Statistical significance was tested by parametric one-way analysis of variance (ANOVA) followed by the Dunnet multiple comparisons test (all groups against the control group) as a post hoc test. Significances are two-sided. Differences were considered statistically significant when p < 0.05.

### *In silico* analysis

A model of DENND5B (Uniprot Q6ZUT9) in complex with RAB39A (Q14964) was generated with ColabFold (https://github.com/sokrypton/ColabFold) with AlphaFold multimer v.3 weights, 48 recycles and a default MMSeqs2 generated MSA, run on one Nvidia A100 slot and 18 CPUs in the UKRI Science and Technology Facilities Council (STFC) Iris cluster. Difference in Gibbs free energy was calculated with PyRosetta and the FastRelax mover (15 cycles, 15 Å neighborhood sphere) following a previous approach.^20^ Interactive model generated with Michelaɴɢʟo.^21^ Data are available at https://github.com/matteoferla/Q6ZUT9-analyses.

## Results

### Identification and *in silico* analysis of *DENND5B* variants

**Figure 1:**
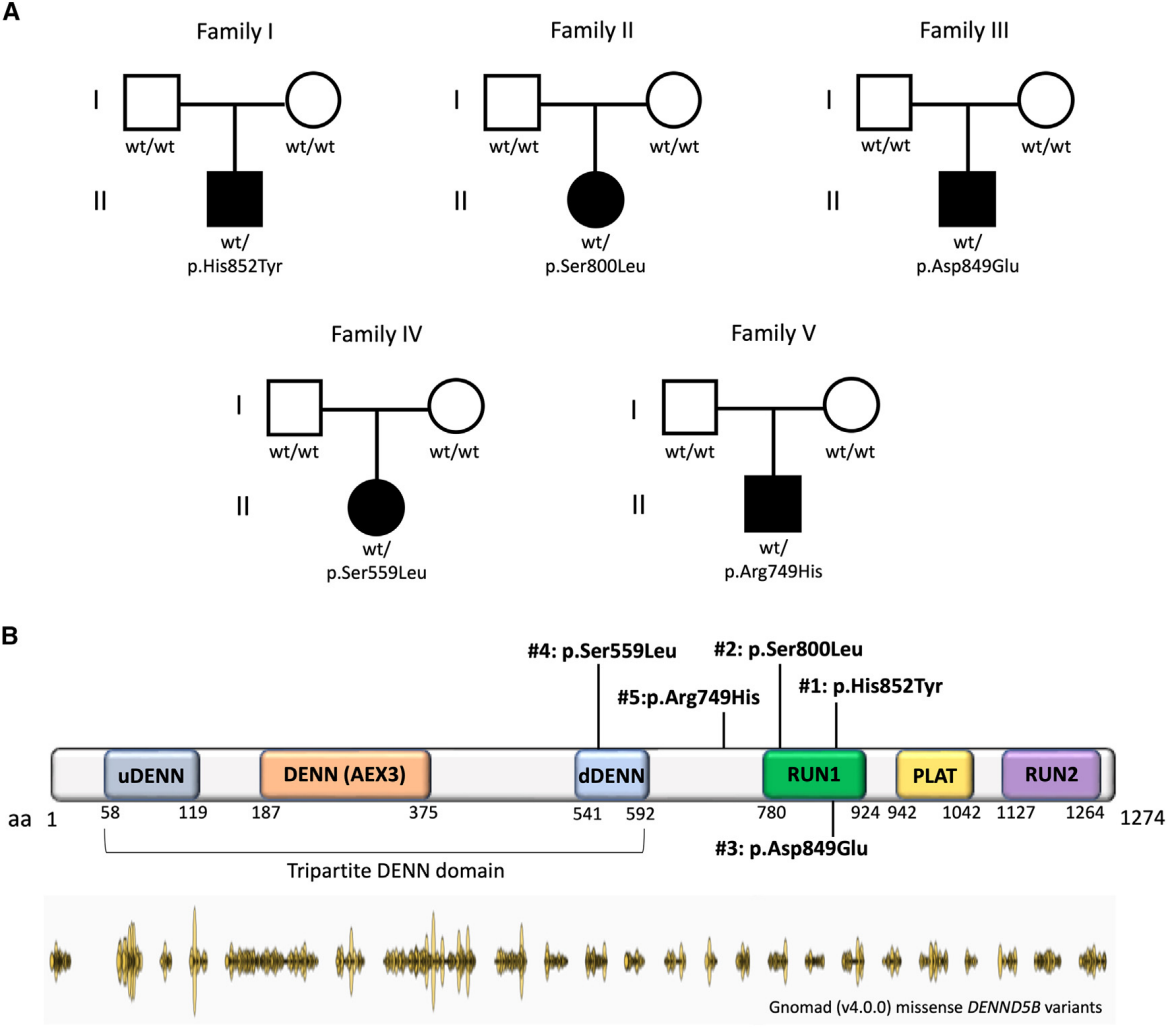
Summary of *DENND5B* variants and family pedigrees (A) Schematic representation of DENND5B (GenBank: NP_659410.3) showing the localization of the variants detected in the reported cohort. Most variants lie within or in close proximity to the tripartite DENN domain or the RUN1 domain. (B) Pedigrees of the reported families showing the segregation of *DENND5B* variants (GenBank: NM_144973.4). Abbreviations: DENN, differentially expressed in neoplastic vs. normal cells; NA, not available; PLAT, polycystin-1, lipoxygenase, alpha-toxin; RUN, RPIP8 (RaP2-interacting protein 8), UNC-14, and NESCA.

ES led to the identification of five distinct heterozygous *DENND5B* variants in the reported individuals: c.2554C>T (p.His852Tyr) in #1; c.2399C>T (p.Ser800Leu) in #2; c.2547C>A (p.Asp849Glu) in #3; c.1676C>T (p.Ser559Leu) in #4; and c.2246G>A (p.Arg749His) in #5. The variants occurred *de novo* in all subjects (Figure 1A). All *DENND5B* variants are rare missense changes (absent in the gnomAD database, v.4.0.0) affecting conserved residues (GERP score 4.05 to 4.99) within or in close proximity to the tripartite DENN domain and, especially, the RUN1 domain (Figure 1B). Four out of five variants are absent in gnomAD (v.4.0.0), whereas the p.Ser800Leu has a very low allele frequency (0.000006574), being reported in a single heterozygous individual. All *DENND5B* variants are predicted to be variably damaging according to several different *in silico* tools (Table S1). No additional potentially deleterious variants in known disease-associated genes were identified in the reported subjects (Table S2). Through gene matching, we also identified two additional potentially deleterious heterozygous variants in *DENND5B* in two subjects showing neurodevelopmental phenotypes, although in the absence of definite segregation information (further details available in the supplemental information, Tables S3 and S4).

### Individuals with *DENND5B* variants display heterogeneous neurodevelopmental phenotypes

**Figure 2:**
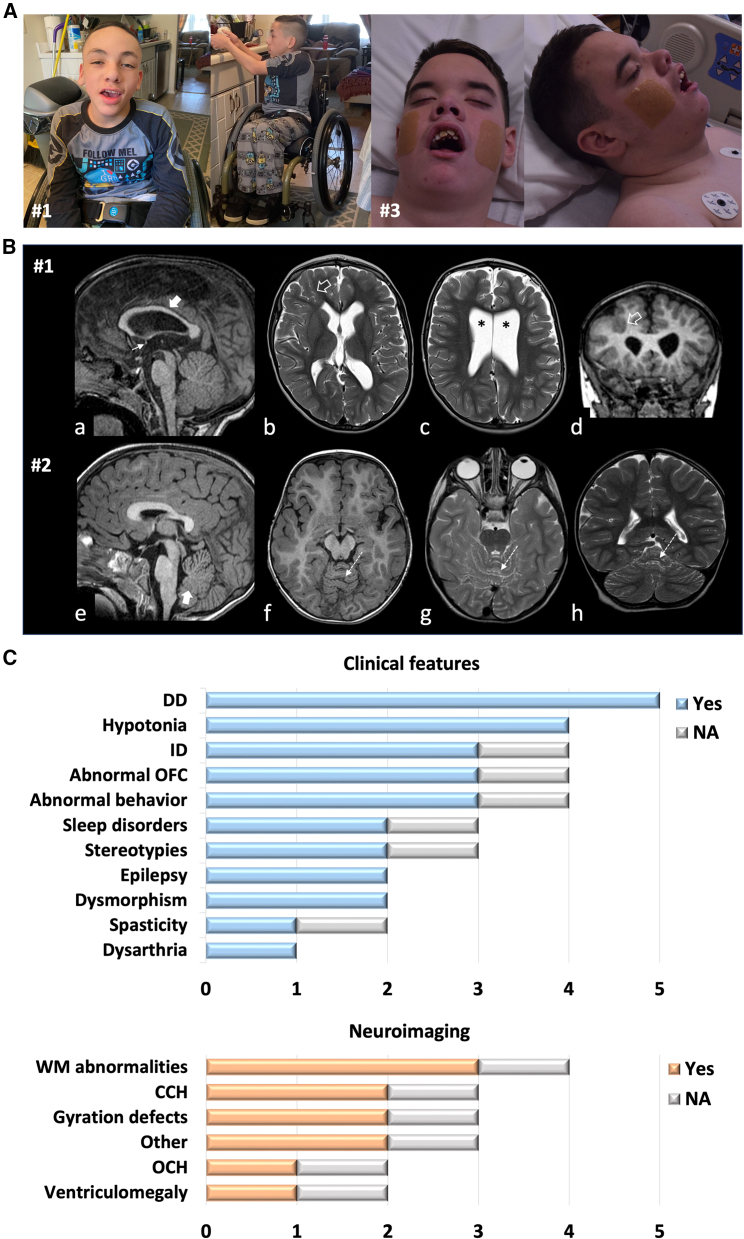
Clinical and neuroimaging details of subjects harboring *DENND5B* variants (A) Clinical photographs of subjects harboring *DENND5B* variants. Subject #1 shows macrocephaly, mild intellectual disability, axial hypotonia, and appendicular spasticity. Facial dysmorphism includes arched and sparse eyebrows, narrow palpebral fissures, hypertelorism, bulbous nose tip, deep nasal bridge, thick lower lip, and prominent chin. Subject #3 has severe cognitive deficiency, microcephaly, and hypotonia. Dysmorphic features include short nose, hypertelorism, malposition of teeth, deep nasal bridge, deep philtrum, and micrognathia. (B) Neuroimaging findings. (a–d) Brain MRI of subject #1 with sagittal T1-weighted (a), axial T2-weighted (b, c), and coronal T1-weighted (d) showing a thin corpus callosum (thick arrow), a small anterior commissure (thin arrow), white matter volume loss with ventricular enlargement (asterisks), and a focal gyral anomaly with infolding in the right frontal lobe (empty arrows). (e–h) Brain MRI of subject #2 with sagittal T1-weighted (a), axial T1-weighted (b), axial T2-weighted (c), and coronal T2-weighted (d) revealing focal foliar anomaly in the superior cerebellar vermis (dashed arrows) associated with inferior vermis hypoplasia (thick arrow). (C) Histogram graphs showing the distribution of clinical features and brain MRI abnormalities in the cohort of subjects harboring *DENND5B* variants. Abbreviations: CCH, corpus callosum hypoplasia; DD, developmental delay; ID, intellectual disability; NA, not available; OCH, optic chiasm hypoplasia; OFC, occipito-frontal circumference; WM, white matter.

Affected individuals harboring *DENND5B* variants showed a clinically heterogeneous neurological involvement, with mild to severe neurodevelopmental phenotypes (Table 1; Figure 2A). All subjects presented with psychomotor delay, leading to variable degrees of intellectual disability (Table 1). Subject #1 showed selective motor delay in the context of spastic/dystonic cerebral palsy, whereas all other subjects were diagnosed with global impairment of psychomotor development. The severity of the developmental delay/intellectual disability phenotype was mild in three subjects (#1, #2, and #5), moderate in one (#4), and severe in one (#3). Of note, no intellectual disability was diagnosed in #2. Overall, this suggests that there is a mixed spectrum of neurodevelopmental disabilities associated with *DENND5B* variants, ranging from mild to severe phenotypes.

**Table 1:** Summary of genetic, clinical, and neuroimaging features in subjects harboring *DENND5B* variants

**Subject ID**	**#1**	**#2**	**#3**	**#4**	**#5**	**Total**
**Age, sex**	**15 years, M**	**12 years, F**	**17 years, M**	**2 years, F**	**15 years, M**	
**Country of origin**	**USA**	**France**	**USA**	**USA**	**USA**	
*DENND5B* variant (NM_144973.4)	c.2554C>T (p.His852Tyr)	c.2399C>T (p.Ser800Leu)	c.2547C>A (p.Asp849Glu)	c.1676C>T (p.Ser559Leu)	c.2246G>A (p.Arg749His)	
Psychomotor delay	+	+	+	+	+	5/5 (100%)
Motor delay	+	+	+	+	+	5/5 (100%)
Speech delay	–	+	+	+	+	4/5 (80%)
Intellectual disability	+	–	+	N/A^a^	+	3/5 (60%)
DD/intellectual disability severity	mild	mild	severe	moderate^a^	mild	
Facial dysmorphism	+	–	+	–	–	
Abnormal OFC	+ (macrocephaly)	+ (macrocephaly)	+ (microcephaly)	N/A	–	3/5 (60%)
Abnormal behavior	+	+	N/A	–	+	3/4 (75%)
ASD	–	+	N/A	–	+	2/4 (50%)
ADHD	+	–	N/A	–	–	1/3 (33.3%)
Stereotyped movements	–	+	N/A	–	+	2/4 (50%)
Neurological features						
Hypotonia	+	–	+	+	+	4/5 (80%)
Spasticity	+ (spastic diplegia)	–	N/A	–	–	1/4 (25%)
Dysarthria	–	–	+	–	–	1/5 (20%)
Sleep disorders	+	–	N/A	–	+	2/4 (50%)
Epilepsy	–	–	+	–	+	2/5 (40%)
Seizure onset	4 years	–	1 year	–	1 year	
Seizure type	febrile, one episode	–	focal	–	multifocal	
EEG	NA	–	FD, SBA	–	MFD, SBA	
Response to AEDs	NA	–	seizure freedom	–	↓ severity	
Brain MRI						
WM involvement	+	–	+	N/A	+	3/4 (75%)
Subcortical abnormalities	+	–	+	N/A	–	2/4 (50%)
CCH	+	–	–	N/A	+	2/4 (50%)
Optic chiasm hypoplasia	–	–	+	N/A	–	1/4 (25%)
Ventricular enlargement	+	–	–	N/A	–	1/4 (25%)
Gyration defects	+	+	–	N/A	–	2/4 (50%)
Other	+	–	+	N/A	–	2/4 (50%)

ADHD, attention deficit-hyperactivity disorder; AEDs, antiepileptic drugs; ASD, autism spectrum disorder; CCH, corpus callosum hypoplasia; DD, developmental delay; F, female; FD, focal discharges; FTT, failure to thrive; ID, intellectual disability; M, male; MFD, multifocal discharges; MFS, multifocal seizures; N/A, not available; OFC, occipito-frontal circumference; SBD, slowing of background activity; WM, white matter; y, years.

a This subject is under five years of age. The degree of intellectual disability is hardly assessable but, based on thorough neuropsychiatric evaluation, the severity of the DD/intellectual disability phenotype was considered as moderate.

Behavioral disorders were present in three subjects. These included attention-deficit hyperactivity disorder (ADHD) in #1, autism spectrum disorder (ASD) in #2 and #5, and a combination of anxiety, compulsiveness, and behavioral dysregulation in #5 (Table S5). Hand stereotypies were observed in two subjects (#2 and #5) and sleep disorders in two individuals (#1 and #5). Four subjects showed hypotonia, either truncal (#1) or generalized (#3-#5). Additional neurological manifestations were heterogeneous and included dysarthria (#3), dystonia (#1), and spasticity (#1). Macrocephaly was present in subjects #1 (2.6 SDS) and #2 (3.19 SDS), whereas microcephaly was observed in individual #3 (−2.48 SDS).

Seizures were reported in three subjects (#1, #3, and #5) (Table S5). While a single episode of febrile seizures occurred in subject #1, recurrent epileptic manifestations were observed in #3 and #5. Subject #3 experienced monthly multifocal seizures and partial seizures with secondary generalization since the age of 1 year. The electroencephalogram (EEG) at onset showed signs of a left anterior temporal partial epilepsy in the context of a slowing of the background activity. Follow-up EEG further showed an involvement of the right frontal region, with features suggestive of a focal cerebral dysfunction. This subject became seizure-free with a combination antiepileptic drug (AED) therapy featuring oxcarbazepine, levetiracetam, zonisamide, clobazam, and lamotrigine. Subject #5 presented with recurrent focal seizures in the first year of life, associated with episodes of behavioral arrest and impaired consciousness. These manifestations typically lasted a few seconds and were characterized by staring and backwards rolling of the eyes.

The EEG showed a diffuse slowing of the background activity, abundant multifocal epileptiform discharges, and brief runs of spike and wave discharges that were particularly evident in the left posterior temporal and right temporal regions. Partial control on seizure severity was achieved with an AED treatment consisting of levetiracetam and valproate, but seizures persisted with weekly episodes.

Brain MRI abnormalities were present in four out of five individuals with an available MRI scan for review (#1, #2, #3, and #5). White matter involvement was noted in three subjects, including volume loss with consequent ventricular enlargement and signal alterations (Figure 2B). Thinning of the corpus callosum was detected in two subjects (#1 and #5). Cortical gyration abnormalities were noted in two subjects, including right frontal focal dysgyria in individual #1 and foliar anomaly in the superior cerebellar vermis in individual #2. Additional findings included hypoplasia of the optic chiasm and bilateral mesial temporal sclerosis in subject #3.

Facial dysmorphism was observed in two subjects (Figure 2A). Individual #1 showed arched and sparse eyebrows, narrow palpebral fissures, hypertelorism, bulbous nose tip, deep philtrum, thick lower lip, and prominent chin. Short nose, hypertelorism, malposition of teeth, deep nasal bridge, deep philtrum, and micrognathia were observed in individual #3. Overall, the overlap was limited to hypertelorism and deep philtrum, thus not supporting an overarching dysmorphism in these individuals. Additional extra-neurological clinical manifestations were also observed in some subjects, involving the visual, cardiac, skeletal, and gastrointestinal systems (Table S5). Although DENND5B has been implicated in the regulation of lipid metabolism and body composition,^12^ no abnormalities in the lipid profile or body fat distribution were detected in our cohort.

### *DENND5B* variants cause decreased protein levels in heterologous expression systems

**Figure 3:**
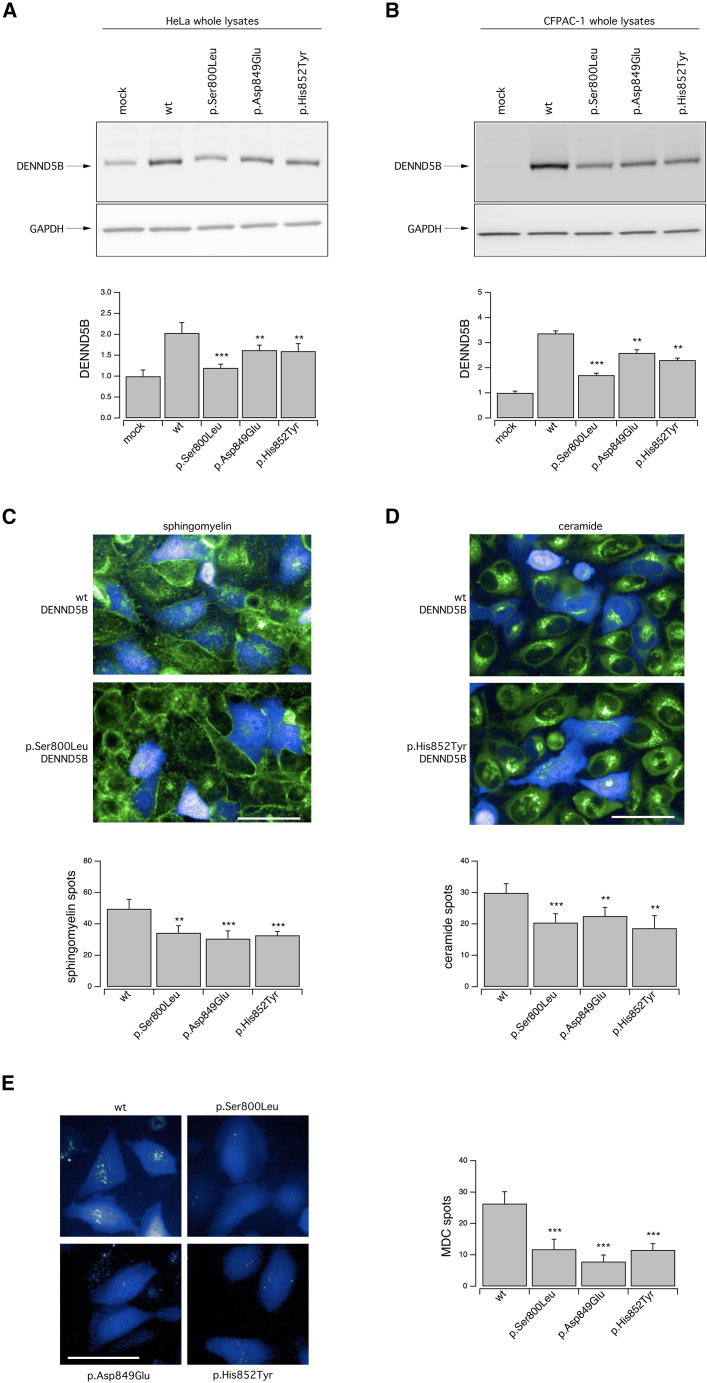
Biochemical and functional analysis of WT and mutant DENND5B proteins in heterologous expression systems DENND5B proteins were transiently expressed in different cell models by means of vectors encoding the different DENND5B proteins and a TagBFP2-based expression marker that allowed easy monitoring of transfection efficiency. (A and B) Western blot analysis (top) and densitometric quantification (bottom) of the levels of the WT, p.Ser800Leu, p.Asp849Glu, and p.His852Tyr DENND5B proteins in whole lysates of HeLa (A) and CFPAC-1 (B) cells. Lysates of mock-transfected parental cells have been included to show the expression level of the endogenous DENND5B protein in the corresponding cell model. Data are means ± SEM (n = 3). (C and D) Representative confocal microscopy images (top) of C12-sphingomyelin (C) or C6-ceramide (D) uptake and quantification (bottom) of fluorescent spots resembling intracellular vesicles containing the corresponding lipid probe. The uptake was assayed on HeLa cells following transient expression of the WT, p.Ser800Leu, p.Asp849Glu, and p.His852Tyr DENND5B constructs. Quantification of spots was performed on TagBFP2-positive cells. Data are means ± SEM (n = 5). For each independent biological replicate, a region measuring 1,320 × 1,320 μm was imaged and analyzed, comprising approximately 35–40 TagBFP2-positive cells. (E) Representative confocal microscopy images (left) and quantification (right) of fluorescent spots resembling autophagic vacuoles visualized using the MDC probe on HeLa cells following transient expression of the WT, p.Ser800Leu, p.Asp849Glu, and p.His852Tyr DENND5B constructs. Quantification of spots was performed on TagBFP2-positive cells. Data are means ± SEM (n = 5). For each independent biological replicate, a region measuring 1,320 × 1,320 μm was imaged and analyzed, comprising approximately 35–40 TagBFP2-positive cells. Asterisks indicate statistical significance vs. control (WT-DENND5B-transfected cells): ^∗∗^p < 0.01; ^∗∗∗^p < 0.001. Scale bar = 100 μm.

We investigated the impact of the detected variants on DENND5B physiological function. To this aim, we selected three variants, p.His852Tyr (in #1), p.Ser800Leu (in #2), and p.Asp849Glu (in #3), affecting the RUN1 domain, where most DENND5B variants were localized. Thse mutants were expressed in different cell models by means of vectors encoding the DENND5B protein (either wild-type or the variant forms) and, as expression marker, an engineered TagBFP2 fused with the neomycin resistance gene *Neo* (TagBFP2/*Neo*) that allowed us to easily monitor transfection efficiency. We expressed WT and DENND5B mutants into two cell lines having different cell background: HeLa (derived from a cervical cancer) and CFPAC-1 (derived from a ductal pancreatic adenocarcinoma). At early timepoints, no difference was observed in the increase over time of the TagBFP2 signal that identifies cells transfected with the different DENND5B forms (Figure S1). We observed that in HeLa cells and CFPAC-1, transfection with the WT-DENND5B construct increased DENNB5B protein accumulation between 2- and 3-fold as compared to the endogenous protein, while the accumulation of mutant forms appeared to be significantly reduced, especially the p.Ser800Leu mutant (Figures 3A and 3B).

### *DENND5B* variants affect lipid uptake and distribution in heterologous expression systems

DENN domain-containing proteins act as GEFs for Rab proteins, thus regulating intracellular organelles and vesicles, and, in turn, membrane trafficking. Based on this observation and the recent evidence that the *Dennd5b*^*−/−*^ mouse model shows a relevant impairment of lipid distribution and metabolism,^12^ we decided to evaluate whether abnormal DENND5B levels led to impaired uptake and distribution of specific lipid probes. To this aim, we exploited high-content confocal imaging and analysis to monitor intracellular uptake and distribution of fluorescent C6-ceramide or C12-sphingomyelin in HeLa cells transiently transfected with the constructs encoding the WT or DENND5B mutants, also bearing a TagBFP2 as expression marker. We therefore measured the intracellular uptake of the fluorescent probes only in cells that were also expressing the BFP, thus overexpressing either the WT or DENND5B mutant proteins. Cells were exposed to the fluorescent lipid probes that were added to the medium and, after 30 min, their uptake and intracellular distribution were evaluated by quantifying, for each BFP-expressing cell, the number of fluorescent spots resembling intracellular vesicles containing fluorescent lipids. Interestingly, we observed that the uptake of both sphingomyelin and ceramide was decreased in cells expressing mutant DENND5B (Figures 3C, 3D, and S2).

We then exploited monodansylcadaverine (MDC), a specific marker for autolysosomes.^22^ When cells are exposed to MDC, the probe is internalized and accumulated inside autophagosomes. Following fusion of autophagosomes with lysosomes, MDC fluorescence increases due to the acidic environment. We therefore quantified, for each BFP-expressing cell, the number of fluorescent MDC spots resembling autophagic vacuoles in HeLa cells overexpressing either the WT or DENND5B mutants (Figure 3E). Resembling what we had observed when assessing internalization and intracellular trafficking of lipid cargoes, the number of MDC-positive spots was markedly reduced in cells expressing DENND5B variants compared to WT (Figure 3E).

### *DENND5B* variants result in decreased protein levels in affected individuals’ fibroblasts

**Figure 4:**
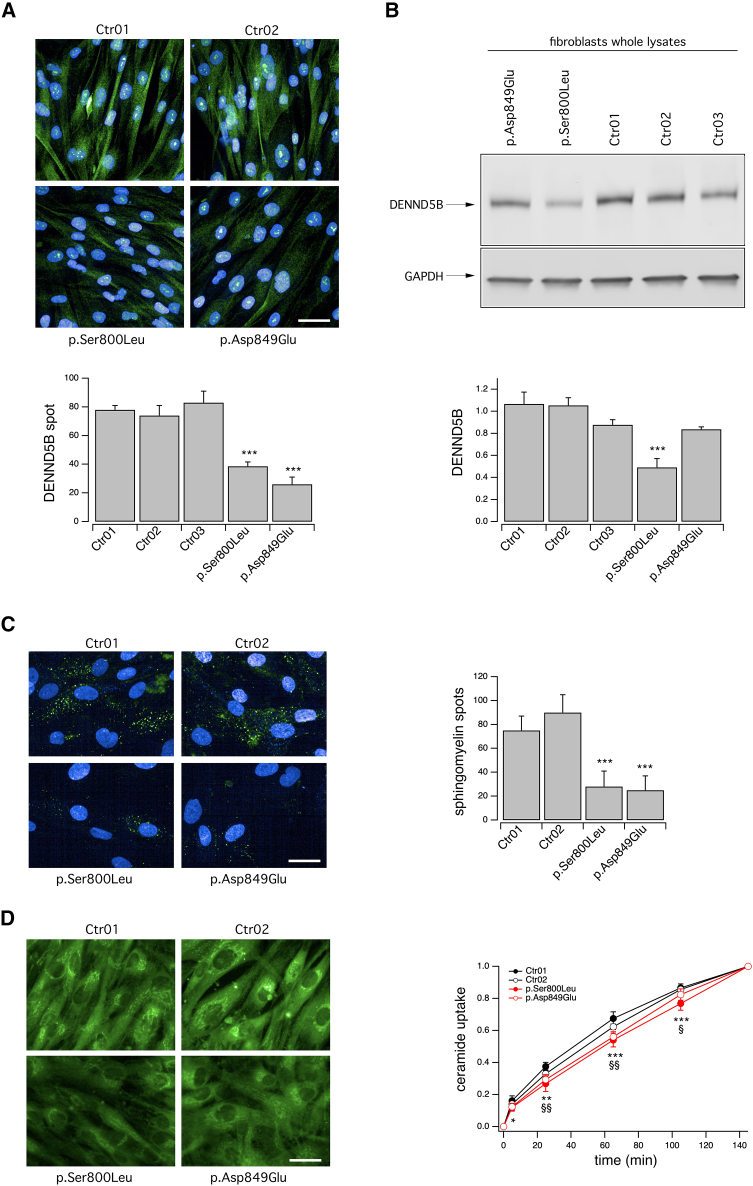
Biochemical and functional characterization of *DENND5B* variants in affected subjects’ fibroblasts (A) Representative confocal microscopy images (top) and quantification (bottom) of DENND5B-positive spots following immunolocalization of DENND5B protein in fibroblasts derived from subjects #2 and #3, carrying, respectively, the p.Ser800Leu and p.Asp849Glu variant and, for comparison, fibroblasts derived from non-NDD subjects. (B) Western blot analysis (top) and densitometric quantification (bottom) of the DENND5B levels in whole lysates of fibroblasts derived from affected individuals and control subjects. Data are means ± SEM (n = 5). (C) Representative confocal microscopy images (left) of C12-sphingomyelin uptake and quantification (right) of fluorescent spots resembling intracellular vesicles containing the lipid probe. The uptake was assayed on fibroblasts derived from affected individuals and control subjects. Data are means ± SEM (n = 9). (D) Representative confocal microscopy images (left) of C6-ceramide uptake and intracellular distribution and its quantification (right). The uptake was assayed on fibroblasts derived from affected individuals and control subjects. Data are means ± SEM (n = 9). For each independent biological replicate, a region measuring 990 × 1,320 μm was imaged and analyzed, comprising approximately one hundred fibroblasts. Asterisks indicate statistical significance: ^∗^p < 0.05; ^∗∗^p < 0.01; ^∗∗∗^p < 0.001 for p.Ser800Leu fibroblasts vs. pool of control fibroblasts; §p < 0.05; §§p < 0.01; for p.Asp849Glu fibroblasts vs. pool of control fibroblasts. Scale bar = 100 μm.

We then isolated fibroblasts from subjects #2 and #3, harboring the p.Ser800Leu and p.Asp849Glu variants, respectively. These changes affect conserved residues within the RUN1 domain, where most of the variants identified in our cohort are localized. For comparison, we utilized already bio-banked fibroblasts derived from four non-NDD donors (i.e., subjects carrying neither DENND5B variants, nor variants in genes related to neurodevelopmental conditions nor, more in general, in genes related to intracellular organelles and vesicles, or involved in membrane trafficking). Immunolocalization of DENND5B revealed that, in fibroblasts derived from affected individuals, the accumulation of the protein was severely decreased, although no changes in its intracellular distribution were detected (Figure 4A). After separation by SDS-PAGE, DENND5B protein level was then analyzed in fibroblast lysates by western blotting, confirming a marked reduction of the p.Ser800Leu mutant accumulation (Figure 4B).

### *DENND5B* variants impair lipid uptake and intracellular distribution dynamics in affected individuals’ fibroblasts

We therefore evaluated the uptake of lipid cargoes by high-content confocal imaging (Figures 4C and 4D). Uptake of C12-sphingomyelin, quantified as the number of fluorescent spots at 30 min after addition of the probe, was significantly impaired in fibroblasts derived from affected individuals (Figure 4C). To further investigate whether DENND5B variants may also impact vesicle trafficking dynamics, we performed a kinetic analysis of fluorescent C6-ceramide intracellular distribution. We demonstrated that not only the uptake but also the time-course distribution of the probe was significantly slowed down in fibroblasts with *DENND5B* variants (Figure 4D).

Then, we wondered whether DENND5B variants may adversely impact the interaction with RAB39A. To this aim, we performed immunolocalization of RAB39 and DENND5B on fibroblasts derived from the enrolled affected individuals and control subjects. Unfortunately, we could not detect any RAB39A signal, suggesting that either the protein is not expressed in fibroblasts, or the antibody does not recognize the specific epitope, despite different commercially available antibodies being used (data not shown). We therefore used a computational approach to model the DENND5B-RAB39A interaction for the WT and variant DENND5B proteins.

### *DENND5B* variants are predicted to disrupt protein folding

**Figure 5:**
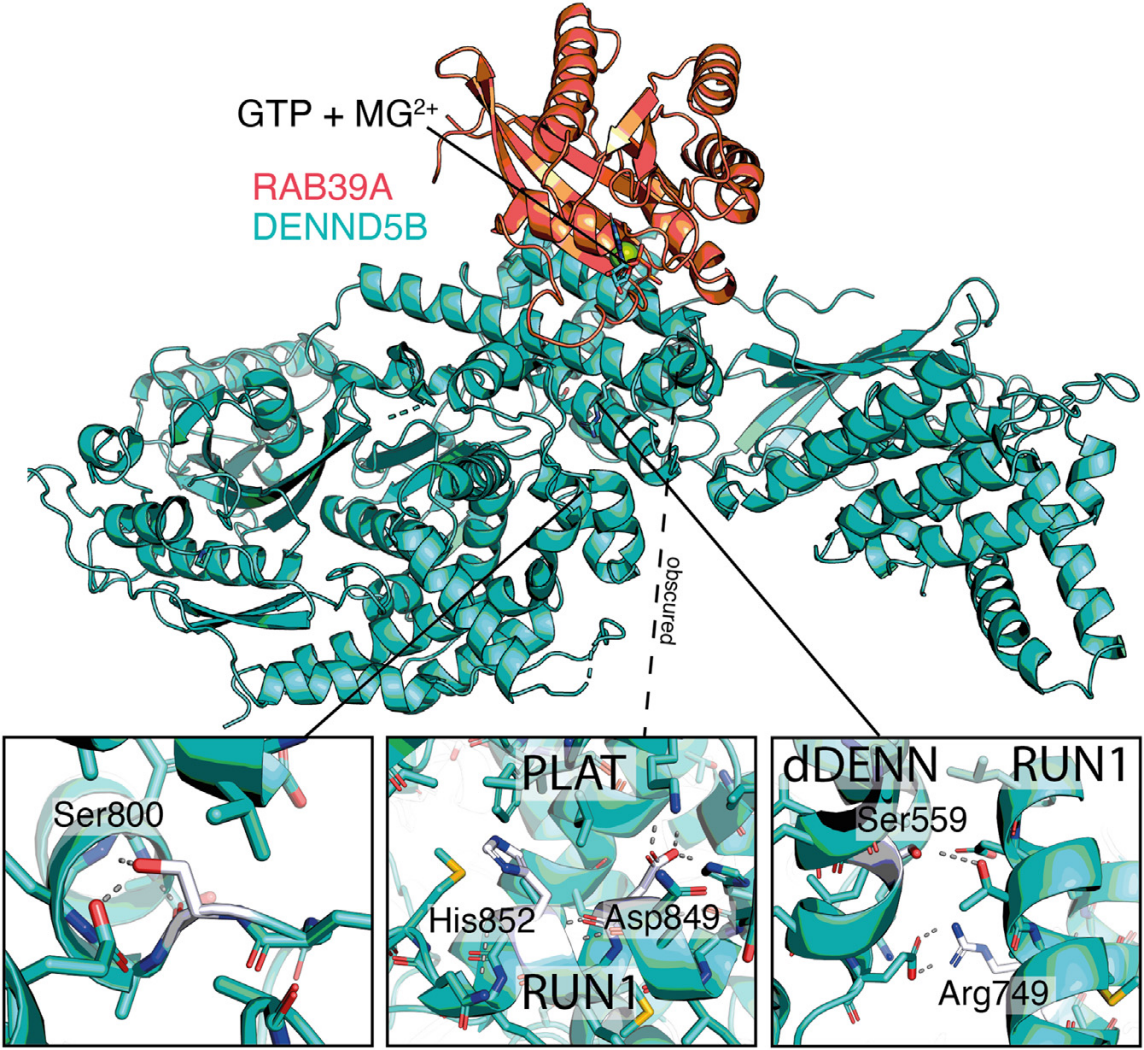
*In silico* analysis of *DENND5B* variants Serine 559 (mutated to leucine in #4) is localized in the dDENN domain. This is a surface mutation facing and in close proximity to the RUN domain, within 5 Å of threonine 776, which is the length of a water bridge. Arginine 749 (histidine 749 in #5) is in the 1st RUN domain. This is a surface variant facing the dDENN domain and act as potentially pathogenic via the same mechanism as p.Ser559Leu. Serine 800 (leucine 800 in #2) is in the 1st RUN domain. This is the last residue in a turn and forms a hydrogen bond with glutamate 786 in the previous helix via its side chain, which would be lost in a leucine variant. In fact, predictions suggest it to be highly destabilizing (>10 kcal/mol). Aspartate 849 (glutamate 849 in #3) is localized in the 1st RUN domain. This buried charged residue forms salt bridges with lysine 876 and histine 882. Despite the similarity of the two residues, glutamate is longer and predictions suggest it to be highly destabilizing (6 kcal/mol). Histidine 852 (tyrosine 852 in #1) is localized in the 1st RUN domain. This buried residue is on the following rung of the helix as aspartate 849 and its side chain appears packed in two alternative conformations depending on the distance to β-hairpin 1015–1019 of the PLAT/LH2 (which shifts by 1 Å). Both conformations form several hydrophobic interactions, but in the more distant conformation (shown), it forms π-π interactions with tryptophan 927 and a π-sulfur interaction with methionine 856, while in the other it forms a hydrogen bond with the backbone of a preceding residue. If this actually represents two biologically relevant states, any change would disrupt this balance.

We generated an *in silico* DENND5B-RAB39A model to assess the potential impact of *DENND5B* variants on this interaction. Models of DENND5 bound to RAB39A generated with ColabFold (AlphaFold2 derivative) had high confidence (plDDT > 0.8) for the variants (Figure 5, interactive at https://michelanglo.sgc.ox.ac.uk/r/dennd5b). Visual inspection and molecular thermodynamics suggest that all the variants disrupt the structure of the protein fold (Figure 5), possibly explaining at least in part the decreased protein accumulation observed in our previous experiments. Intriguingly, except for the highly destabilizing p.Ser800Leu, the variants are found at the interfaces between domains. The p.Ser559Leu and p.Arg749His variants are 8 Å away on the different sides of the dDENN and RUN1 domain interface, while p.Asp849Glu and p.His852Tyr are in the same direction on the same helix at the interface between the RUN1 and PLAT domains. None of the variants are directly located at the interface with the RAB GTPase. In the five predicted models, two different packing solutions for this interface are seen with the opposite domain shifting by 1 Å. Given the very high confidence of these residues (>0.9), it cannot be ruled out that these are two biologically relevant states reflecting active or inactive guanine nucleotide exchange states.

## Discussion

In this study, we describe a complex neurodevelopmental disorder associated with heterozygous *de novo* variants in *DENND5B*, encoding a GEF relevant to the activation of Rab family proteins, which are crucial regulators of intracellular vesicular trafficking.^1^^,^^2^ Affected individuals presented with mild cognitive impairment, behavioral disorders, and a spectrum of brain MRI abnormalities featuring white matter involvement and cortical gyration defects. Facial dysmorphism and heterogeneous extra-neurological manifestations were observed in some affected individuals, featuring developmental defects involving the visual, cardiac, skeletal, and gastrointestinal systems. Functional investigation of *DENND5B* variants demonstrated that these non-synonymous changes affect protein accumulation, possibly due to an impairment of protein folding, and disrupt lipid uptake and intracellular distribution, also affecting the dynamics of vesicle trafficking.

DENND5B interacts with different members of the Rab family, including Rab6 and Rab39.^8^ Variants in genes encoding Rab proteins are implicated in the pathogenesis of several human diseases, including neurological disorders.^3^^,^^23^ Recurrent *de novo* loss-of-function variants in *RAB11B* (MIM: 604198) leading to altered GDP/GTP binding cause a neurodevelopmental syndrome with epilepsy, ataxia, abnormal vision, and musculoskeletal abnormalities (NDAGSCW [MIM: 617807]).^24^ DENND5B does not directly interact with RAB11B. However, some clinical manifestations observed in the subjects enrolled in our study are crucial features of NDAGSCW, such as cerebellar involvement and musculoskeletal defects.^24^ Loss-of-function variants in *RAB39B* (MIM: 300774) may result in a similar neurodevelopmental disorder (XLID72 [MIM: 300271]) or in a distinct condition with intellectual disability and early-onset Parkinsonism (Waisman syndrome [MIM: 311510]).^25^^,^^26^^,^^27^ Subjects harboring *DENND5B* variants show clinical features common to these two conditions, especially XLID72, suggesting that the disruption of DENND5B-Rab39 interaction may contribute to the pathogenesis of the *DENND5B*-related neurodevelopmental disorder. The presence of gyration defects is an interesting MRI feature of subjects harboring *DENND5B* variants, since the proper orchestration of vesicle trafficking is crucial for the development of the nervous system.^28^ Indeed, among other effects, vesicle-mediated signaling defects may impact the migration of neuronal progenitors during cortical development and alter oligodendrocyte biology.^29^

The *DENND5B* variants detected in our cohort are predicted to disrupt protein folding and to impair DENND5B-mediated lipid uptake and distribution. *DENND5B* shows a high intolerance to missense changes (observed/expected variants ratio = 0.63, range 0.58–0.68) and loss of function (observed/expected variants ratio = 0.07, range 0.03–0.15).^30^ These observations support the association between loss-of-function *DENND5B* variants and the neurodevelopmental phenotypes observed in the affected individuals enrolled in our study. The missense changes detected in our cohort affect conserved residues within functional domains of DENND5B (Figure 1B). Three variants (p.Ser800Leu, p.Asp849Glu, and p.His852Tyr) lie contiguously within RUN1, whereas the p.Arg749His variant is localized in close proximity of this domain. p.Ser559Leu affects a conserved residue within dDENN (downstream DENN). Both RUN1 and dDENN are crucial for protein function but, interestingly, they are not depleted of missense changes according to gnomAD (v.4.0.0) (Tables S6 and S7). Thus, it is possible that the involvement of specific residues within these domains causes recognizable cellular and clinical phenotypes. For example, p.Ser800Leu led to a marked decrease in protein levels compared to other changes and was associated with a distinctive gyration defect (Table 1). Interestingly, this same variant is present in a single heterozygous individual in gnomAD (v.4.0.0), suggesting the possibility of incomplete penetrance or the association with a milder neurological phenotype. Future studies will be crucial to dissect whether *DENND5B* variants lead to a complete loss of protein function or, rather, act as hypomorphs.

As the majority of *DENND5B* missense changes detected in our cohort affected the RUN1 domain, we investigated variants in this domain using different cellular approaches. Western blot and immunolocalization analyses revealed decreases of protein accumulation in both heterologous systems and fibroblasts obtained from affected subjects. This finding may be at least partially explained by a misfolding defect and subsequent premature degradation mediated by the proteasome, as suggested by the *in silico* analysis performed on our DENND5B-RAB39A model. Functional investigation of the variants demonstrated that the reduced protein levels led to a significant impairment of intracellular uptake and distribution of lipid cargoes, supporting a role of DENND5B in the regulation of the endosomal system and intracellular membrane trafficking pathways. Although further confirmation is needed, we also demonstrated that DENND5B deficiency causes a slowdown of these processes, suggesting that DENND5B activity is important to maintain the appropriate dynamics of vesicle trafficking. Additionally, MDC-based assay revealed a decreased number of autophagic vacuoles in mutant cells, suggesting that DENND5B deficiency may impact autophagy pathways. The processes affected by DENND5B deficiency are crucial for many functions in neuronal cells, including axonal transport, synaptic function, and synaptic plasticity.^7^^,^^16^^,^^17^ Thus, it is possible that the *DENND5B* variants detected in the subjects enrolled in our study cause significant neuronal functional damage, accounting for the observed neurodevelopmental and epileptic phenotypes.

Abnormal head circumference is present in different neurodevelopmental conditions, including disorders caused by variable defects in vesicle trafficking pathways.^31^^,^^32^^,^^33^ Microcephaly secondary to abnormal proliferation of neural progenitors has been directly associated with endosomal trafficking defects, whereas macrocephaly is part of the phenotype of many NDDs caused by the dysfunction of vesicle trafficking regulators, such as *RAC1*- (MIM: 602048) and *RAB39B*-related disorders.^25^^,^^33^^,^^34^^,^^35^^,^^36^^,^^37^ The presence of either microcephaly (subject #3) or macrocephaly (subjects #1 and #2) in our cohort may be the result of the different functional impact of DENND5B variants. In addition to a shared deleterious effect on protein levels, the variants identified in these subjects might affect RUN1 functions in the residual portion of functional protein in a variant-specific manner. For example, RAB6 controls the dynamic balance between the formation and turnover of autophagosomes, and loss of RAB6 results in a significant reduction of cell size.^38^ Thus, a more or less severe impairment of DENND5B-RAB6 interaction might contribute to the different brain size in subjects #1, #2, and #3. During brain development, autophagy plays a crucial role in the fine regulation of neurogenesis, neuronal maturation, and cell survival.^39^^,^^40^^,^^41^^,^^42^ These events are crucial for brain growth and microcephaly is common in neurodevelopmental conditions caused by autophagy dysregulation.^43^^,^^44^ Our MDC-based assay showed that *DENND5B* variants impact on autophagy-related processes, suggesting that autophagy defects may contribute to determine brain size in subjects harboring *DENND5B* variants. Comprehensively, different *DENND5B* variants might lead to the disruption of specific functional pathways, underlying distinctive patterns of abnormal neurodevelopment and cranial growth defects.

Epileptic manifestations were observed in two affected individuals in our cohort. DENND5B levels were found to be reduced in the brains of subjects with temporal lobe epilepsy and in two distinct chronic epileptic mouse models (kainic acid [KA] and pentylenetetrazole [PTZ] kindling mice), suggesting that *DENND5B* expression correlates with epileptogenesis.^7^ *Dennd5b* knockdown leads to increased epileptic discharge and reduced incubation period, whereas its overexpression results in the opposite phenotype.^7^ Interestingly, epileptic manifestations are part of the core phenotype of XLID72 and DEE49, two conditions showing a certain overlap with *DENND5B*-related disorder.^16^^,^^25^ DENND5 proteins also interact with SNX1, which is implicated in the regulation of the physiological trafficking of the group I metabotropic glutamate receptors (mGluRs) receptors (mGluR1 and mGluR5).^10^ These receptors are crucial for synaptic plasticity and their dysfunction may lead to intellectual disability, epilepsy, and ASD.^45^ Interacting with RAB6 and DENND5 proteins, the N terminus of SNX1 can determine vesicle specificity, while the C-terminal PX (Phox-homology) and BAR (Bin, amphiphysin, and Rvs) domains exert the lipid attachment.^9^ Thus, an abnormal DENND5B-SNX1 binding might disrupt vesicle transport between early endosomes and TGN, possibly affecting synaptic function and determining increased epileptogenesis.^10^

The *DENND5B* variants identified in our cohort segregated with the clinical phenotypes in a dominant manner. This is uncommon in human disorders resulting from dysregulated GEF activity, which mostly occur in the presence of bi-allelic deleterious variants.^16^^,^^46^ However, rare dominant conditions have been also reported. For example, heterozygous loss-of-function variants in *FLCN* (MIM: 607273) cause a complex genodermatosis known as Birt-Hogg-Dube syndrome (MIM: 135150).^47^ *FLCN* encodes folliculin, a regulator of vesicle trafficking that interacts with Rab GTPases and contains a C-terminal DENN-like domain.^48^ Although Folliculin acts as a GEF for RAB35, it also activates other Rabs using different mechanisms.^49^^,^^50^^,^^51^ It facilitates the binding of RAB34 to its effector RILP and the loading of PAT1, an important regulator of early endocytosis, on RAB11A.^50^^,^^51^ Resembling *FLCN*, dominant *DENND5B* variants could be deleterious through different mechanisms, regardless of a decreased GEF activity. This is also suggested by their enrichment in the RUN1 domain. *DENND5B* variants could disrupt DENND5B-Rabs interactions, impairing the binding to crucial effectors with a regulatory role in vesicle trafficking.^1^^,^^2^^,^^46^^,^^51^ The haploinsufficiency model in *DENND5B*-related disorder is also supported by the observation that the lentiviral-mediated knockdown of *Dennd5b* in mouse results in a roughly 50% decrease of functional protein, leading to a severe epileptic phenotype.^7^ Conversely, *DENND5B* polymorphisms associated with lipid homeostasis abnormalities follow a recessive inheritance pattern and are predicted to result in mild deleterious effects (Table S8). Thus, these polymorphisms might act through different mechanisms in the context of a predisposing genetic background.^12^^,^^13^^,^^14^^,^^15^ Overall, a single functional allele of *DENND5B* may not be sufficient to guarantee the physiological activity of the protein, supporting a disease model where heterozygous loss-of-function variants cause abnormal membrane trafficking and neurodevelopmental disorder.

In conclusion, we report a complex neurodevelopmental condition featuring cognitive impairment, variable epilepsy, and brain abnormalities in association with heterozygous *de novo* loss-of-function variants in *DENND5B*. Biochemical studies confirmed that *DENND5B* variants result in a decreased protein levels, and the functional investigation of the variants demonstrated a significant impact on the intracellular lipid metabolism and vesicle transport. Collectively, our findings support the role of loss-of-function variants in *DENND5B* as the cause of a distinctive neurodevelopmental condition, highlighting the crucial conserved role of DENND5B in brain development and reinforcing the association of DENND5B deficiency with increased epileptogenesis in human subjects.

## Data and code availability

The data used to support the conclusions in this paper are available from the corresponding author upon reasonable request. *In silico* data analysis is available at https://github.com/matteoferla/Q6ZUT9-analyses.

All variants were submitted to the Leiden Open Variation Database (LOVD) and ClinVar. The accession numbers for the DENND5B variants reported in this paper are LOVD: #0000932979, #0000932981, #0000932982, #0000932983, #0000932984, #0000932985, and #0000932986. The accession numbers for the *DENND5B* variants reported in this paper are ClinVar: VCV002664959.1, VCV002664958.1, VCV002664957.1, VCV002664956.1, and VCV002664955.1.

## Web resources

ClinVar, https://www.ncbi.nlm.nih.gov/clinvar
Combined Annotation Dependent Depletion (CADD), http://cadd.gs.washington.edu
DECIPHER, https://decipher.sanger.ac.uk
Ensembl Variant Effect Predictor (VEP), https://www.ensembl.org/info/docs/tools/vep/index.html
GenBank, https://www.ncbi.nlm.nih.gov/genbank/
Gene Cards, https://www.genecards.org
Gene Matcher, http://www.genematcher.org
Genome Aggregation Database (GnomAD), http://gnomad.broadinstitute.org
Genomic Evolutionary Rate Profiling (GERP), http://mendel.stanford.edu/SidowLab/downloads/gerp/
Mutation Taster, http://www.mutationtaster.org
NeXtProt, https://www.nextprot.org
Online Mendelian Inheritance in Man, https://www.ncbi.nlm.nih.gov/Omim
PubMed, https://www.ncbi.nlm.nih.gov/pubmed
RefSeq, https://www.ncbi.nlm.nih.gov/refseq
SIFT, https://sift.bii.a-star.edu.sg
UniProt, https://www.uniprot.org
UCSC Human Genome Database, https://www.genome.ucsc.edu
Varsome, https://varsome.com

## Supplemental information

Document S1. Figures S1 and S2 and supplemental methods

Table S1. *In silico* analysis of DENND5B variants

Table S2. Additional variants identified in DENND5B patients

Table S3. *In silico* analysis of the two additional potentially deleterious DENND5B variants

Table S4. Clinical features of the two additional subjects harboring potentially delterious DENND5B variants

Table S5. Clinical features of DENND5B patients

Table S6. Missense variants in DENND5B according to gnomAD

Table S7. Missense variation of DENND5B domains

Table S8. *In silico* analysis of DENND5B polymorphisms reported in the literature

Document S2. Article plus supplemental information
