# Supplementary material for: De novo variants in DENND5B cause a neurodevelopmental disorder
Source: Am J Hum Genet. 2024 Feb 21;111(3):529–43. doi: 10.1016/j.ajhg.2024.02.001 (PMC10940048; doi:10.1016/j.ajhg.2024.02.001)
Supplement: Document S1. Figures S1 and S2 and supplemental methods [file mmc1.pdf]

The American Journal of Human Genetics, Volume *111*

# Supplemental information

# *De novo* variants in *DENND5B*

# cause a neurodevelopmental disorder

**Marcello Scala, Valeria Tomati, Matteo Ferla, Mariateresa Lena, Julie S. Cohen, Ali Fatemi, Elly Brokamp, Anna Bican, John A. Phillips III, Mary E. Koziura, Michael Nicouleau, Marlene Rio, Karine Siquier, Nathalie Boddaert, Ilaria Musante, Serena Tamburro, Simona Baldassari, Michele Iacomino, Paolo Scudieri, Undiagnosed Diseases Network, Jill A. Rosenfeld, Gary Bellus, Sara Reed, Hind Al Saif, Rossana Sanchez Russo, Matthew B. Walsh, Vincent Cantagrel, Amy Crunk, Stefano Gustincich, Sarah M. Ruggiero, Mark P. Fitzgerald, Ingo Helbig, Pasquale Striano, Mariasavina Severino, Vincenzo Salpietro, Nicoletta Pedemonte, and Federico Zara**

**Supplemental Material**

**1. Supplemental Methods**

**2. Supplemental Results**

**3. Supplemental Tables**

**4. Supplemental Figures**

**5. Supplemental References**

**6. Supplemental information**

## 1. Supplemental Methods

### *1.1 Participants enrolment*

The patients investigated in this study were enrolled at several different international research centers and hospitals: Developmental Brain Disorders Laboratory, Imagine Institute, INSERM, Paris, France; Emory University, Atlanta, GA 30322, USA; Geisinger Medical Center, Danville, PA, USA; Kennedy Krieger Institute, Baltimore, Maryland, USA; Vanderbilt University Medical Center, Nashville, Tennessee, USA; Virginia Commonwealth University School of Medicine, Richmond, Virginia, USA.

### *1.2 Exome sequencing analysis*

For exome sequencing (ES), genomic DNA was extracted from peripheral blood lymphocytes or buccal swab samples of the probands and parents (when available). After standard DNA extraction, exome sequencing (ES) (Trio-ES in subjects #1-4 and #7; proband-only ES in #5 and #6) was performed as previously described (Aspromonte et al., 2019; Bowling et al., 2017; Harripaul et al., 2017; Murdock et al., 2021; Tarailo-Graovac et al., 2016; Guillen Sacoto et al., 2020). QC statistics with FastQC (http://www.bioinformatics.bbsrc.ac.uk/projects/fastqc) was used to assess the quality of the sequence reads. BWA with default parameters was used for reads alignment to the reference human genome (GRCh38 - hg38, UCSC genome assembly). Recalibration of the quality score and for indel realignment and variant calling was performed through the HaplotypeCaller algorithm within the GATK package (DePristo et al., 2011; McKenna et al., 2010). Variants were then annotated with ANNOVAR (Wang et al., 2010) and filtered out for minor allele frequency (MAF) ≤ 0.01 in genomic databases (GnomAD v4.0.0, https://gnomad.broadinstitute.org). Afterwards, *in silico* tools were employed to predict the impact of candidate variants on protein structure and function, including: Combined Annotation Dependent Depletion (CADD, https://cadd.gs.washington.edu), Mutation Taster (http://www.mutationtaster.org), Mutation Assessor (http://mutationassessor.org/r3/), Polyphen-2 (http://genetics.bwh.harvard.edu/pph2/), and

Splice AI (https://spliceailookup.broadinstitute.org). Sanger sequencing was performed according to standard procedures (Tarailo-Graovac et al., 2016) to confirm the most plausible candidate variants and for parental segregation analysis.

### *1.3 Copy Number Variations analysis*

For the detection of copy number variations (CNVs), array comparative genomic hybridization (Array-CGH) (Agilent 60K) was performed as previously described in all subjects except patient #1 (Redon et al., 2009). In this individual, CNVs calling was performed using a custom-developed analysis tool (XomeAnalyzer). Data were filtered and analyzed to identify sequence variants and most deletions and duplications involving three or more coding exons, as previously described (Retterer et al., 2015). Potential rearrangements were interpreted according to the Decipher database (https://decipher.sanger.ac.uk).

## 2. Supplemental Results

*Supplemental genetic findings in DENND5B patients*

Additional genetic variants were detected in four patients. In subject #1, an interstitial duplication in chromosome 2 was identified: arr[GRCh37] 2p14(64217422_65650528) x3. This rearrangement was confirmed by FISH and parental FISH testing showed that the mother harbored the same duplication. The duplicated region does not contain any genes associated with autosomal dominant disorders or known pathogenic CNVs. Furthermore, the mother of the patient was completely asymptomatic. Thus this duplication is unlikely to be contributing to his phenotype and can be classified as uncertain significance. In subject #4, the paternally inherited *POLR3A* (NM_007055.4): c.2350G>A (p.Gly784Ser) variant was identified. This variant is reported in gnomAD with an allele frequency of 0.00000979, being observed in two individuals in heterozygous state, and it is predicted pathogenic according to *in silico* tools (PM2, PP3, and PP5 criteria). Biallelic variants in *POLR3A* (Polymerase III, RNA, subunit a, MIM * 614258) cause

Leukodystrophy, hypomyelinating, 7, with or without oligodontia and/or hypogonadotropic hypogonadism (MIM # 607694) or Wiedemann-Rautenstrauch syndrome (MIM # 264090). However, the phenotype of this patient is not consistent with these conditions and no additional variants could be detected in *POLR3A*. In subect #5, the maternally inherited *USP9X* (NM_001039591.3): c.367G>T (p.Gly123Trp) variant was detected. This amino acid change is absent in gnomAD and has conflicting predictions of pathogenicity by *in silico* tools. According to the ACCMG-AMP criteria (BP1, PM2, PP3), this variant is classified as a variant of uncertain significance (VUS). Genetic variants in the *USP9X* gene (Ubiquitin-specific protease 9, X-linked; MIM * 300072) cause a recessive (Intellectual developmental disorder, X-linked 99, XLID99, MIM # 300919) and a dominant (Intellectual developmental disorder, X-linked 99, syndromic, female-restricted, MRXS99F, MIM # 300968) form of X-linked intellectual disability. However, this patient did not show dysmorphic or neuropsychiatric features suggestive of XLID99.

*Identification of additional subjects harboring potentially deleterious DENND5B variants*

While collecting patients through gene matching platforms, we also came across two additional individuals harboring potentially deleterious heterozygous variants in the *DENND5B* gene and presenting with neurodevelopmental phenotypes that partially overlap the clinical manifestations observed in our cohort (Table S4). These two patients (subjects #6 and #7) presented with developmental delay, dysmorphism, and white matter involvement on brain MRI. They harbored the missense variant c.709C>T, p.(Pro237Ser) (subject #6) and the splicing variant c.237+3A>G (subject #7). These variants are rare and predicted to be likely damaging according to *in silico* tools (Table S5). The p.(Pro237Ser) variant detected in subject #6 affects a conserved residue within the DENN (AEX) domain, whereas the c.237+3A>G variant detected in #7 involves a donor site in intron 2 of 20 and might affect splicing (scSNV-ADA = 0.970115). This splicing variant is predicted to disrupt splicing, leading to the formation of either a truncated transcript or a nonsense mediated mRNA decay (NMD).

*Supplemental genetic findings in the subjects with potentially deleterious DENND5B variants*

In subject #6, ES detected the NC_012920(MT-TA):m.5590G>A in heteroplasmic status (52%), which was interpreted as a variant of unknown significance. In subject #7, ES led to the identification of the *COL11A1* variants (NM_001854.3): c.2921C>G, (p.Pro974Arg) and c.4534A>G, (p.Lys1512Glu), of unknown inheritance. Autosomal recessive variants in the *COLL11A* gene (MIM * 120280) are associated with Fibrochondrogenesis type 1, a complex condition characterized by facial dysmorphism and extensive skeletal defects (Guo et al., 2017). Similar clinical features could not be observed in this patient.

## 3. Supplemental Tables

**Table S1. *In silico* analysis of *DENND5B* variants.**

**Table S2. Additional variants identified in DENND5B patients.**

**Table S3. *In silico* analysis of the two additional potentially deleterious *DENND5B* variants.**

**Table S4. Clinical features of the two additional subjects harboring potentially delterious *DENND5B* variants.**

**Table S5. Clinical features of *DENND5B* patients.**

**Table S6. Missense variants in DENND5B according to gnomAD.**

**Table S7. Missense variation of DENND5B domains.**

**Table S8. *In silico* analysis of *DENND5B* polymorphisms reported in the literature.**

## 4. Supplemental Figures

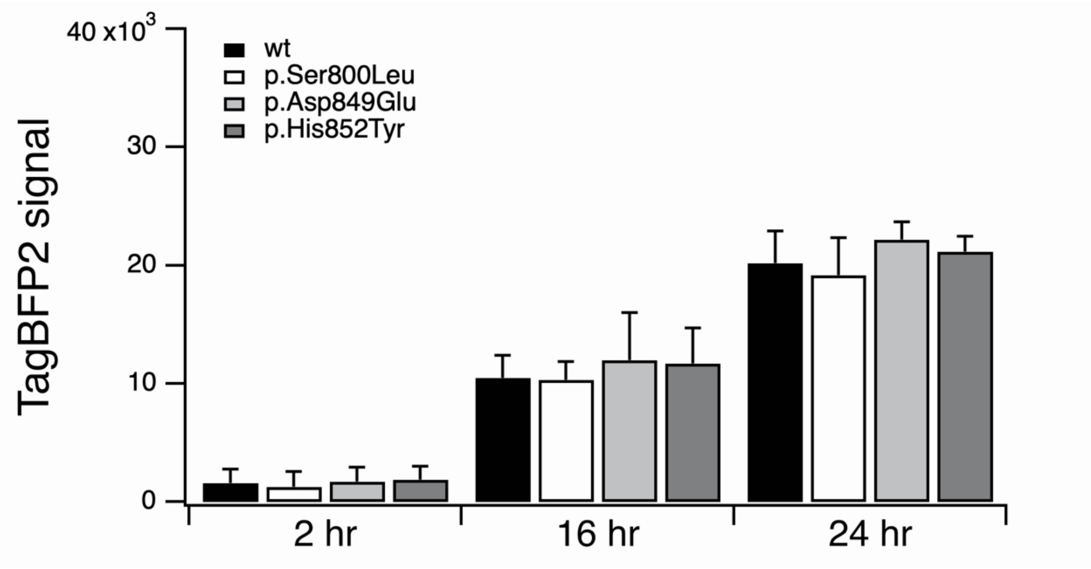


**Supplemental Figure S1. Analysis of heterologous expression of wild-type and variant DENND5B proteins in HeLa cells.** DENND5B proteins were transiently expressed in HeLa cells by means of vectors encoding the different DENND5B proteins followed by the TagBFP2. TagBFP2 fluorescence was monitored from 2 hr up to 24 hr after transfection at the indicated timepoints. The observed increase over time of the TagBFP2 signal (that identifies cells transfected with the different DENND5B forms) was comparable, suggesting no significant differences in the transcription of the transgene.

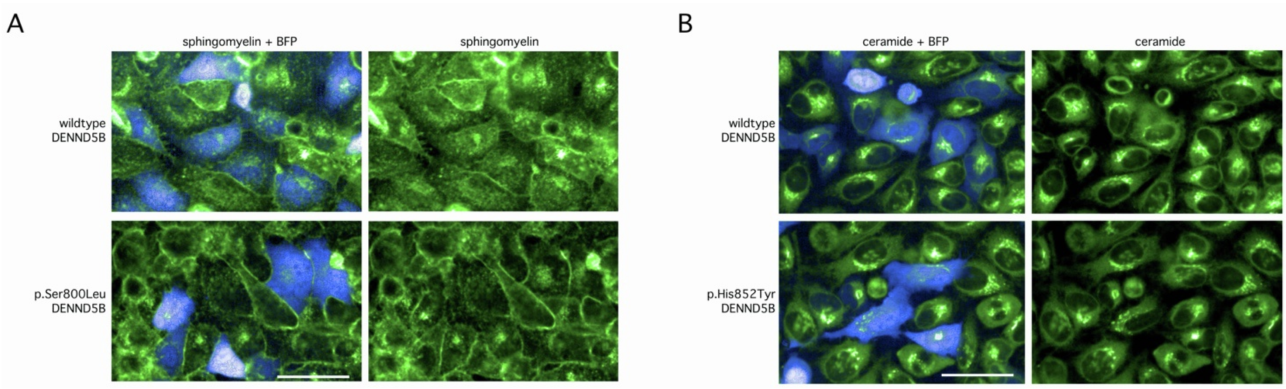


**Supplemental Figure S2. Functional analysis of sphingolipids uptake in HeLa cells expressing wild-type and variant DENND5B proteins.** DENND5B proteins were transiently expressed in HeLa cells by means of vectors encoding the different DENND5B proteins and a TagBFP2-based expression marker that allowed easy monitoring of transfection efficiency. A-B) Confocal microscopy images reported in Figure 3C,D and showing of C12-sphingomyelin (A) or C6-ceramide (B) fluorescent spots alone (green signal, right panels) or merged with the TagBFP2 signal (left panels) that identifies cells transfected with the different DENND5B forms. Scale bar = 100 μm.

**5. Supplemental References**


Aspromonte MC, Bellini M, Gasparini A, Carraro M, Bettella E, Polli R, Cesca F, Bigoni S, Boni S, Carlet O, Negrin S, Mammi I, Milani D, Peron A, Sartori S, Toldo I, Soli F, Turolla L, Stanzial F, Benedicenti F, Marino-Buslje C, Tosatto SCE, Murgia A, Leonardi E. Characterization of intellectual disability and autism comorbidity through gene panel sequencing. Hum Mutat. 2019 Sep;40(9):1346-1363. doi: 10.1002/humu.23822. Epub 2019 Aug 2. Erratum in: Hum Mutat. 2020 Jun;41(6):1183.

Bowling KM, Thompson ML, Amaral MD, Finnila CR, Hiatt SM, Engel KL, Cochran JN, Brothers KB, East KM, Gray DE, Kelley WV, Lamb NE, Lose EJ, Rich CA, Simmons S, Whittle JS, Weaver BT, Nesmith AS, Myers RM, Barsh GS, Bebin EM, Cooper GM. Genomic diagnosis for children with intellectual disability and/or developmental delay. Genome Med. 2017 May 30;9(1):43. doi: 10.1186/s13073-017-0433-1.

DePristo MA, Banks E, Poplin R, et al. A framework for variation discovery and genotyping using next-generation DNA sequencing data. Nat Genet. 2011;43:491–8.

Guillen Sacoto MJ, Tchasovnikarova IA, Torti E, Forster C, Andrew EH, Anselm I, Baranano KW, Briere LC, Cohen JS, Craigen WJ, Cytrynbaum C, Ekhilevitch N, Elrick MJ, Fatemi A, Fraser JL, Gallagher RC, Guerin A, Haynes D, High FA, Inglese CN, Kiss C, Koenig MK, Krier J, Lindstrom K, Marble M, Meddaugh H, Moran ES, Morel CF, Mu W, Muller EA 2nd, Nance J, Natowicz MR, Numis AL, Ostrem B, Pappas J, Stafstrom CE, Streff H, Sweetser DA, Szybowska M; Undiagnosed Diseases Network; Walker MA, Wang W, Weiss K, Weksberg R, Wheeler PG, Yoon G, Kingston RE, Juusola J. De Novo Variants in the ATPase Module of MORC2 Cause a Neurodevelopmental Disorder with Growth Retardation and Variable Craniofacial Dysmorphism. Am J Hum Genet. 2020 Aug 6;107(2):352-363. doi: 10.1016/j.ajhg.2020.06.013. Epub 2020 Jul 20. PMID: 32693025; PMCID: PMC7413887.

Guo L, Elcioglu NH, Wang Z, Demirkol YK, Isguven P, Matsumoto N, Nishimura G, Miyake N, Ikegawa S. Novel and recurrent COL11A1 and COL2A1 mutations in the Marshall-Stickler syndrome spectrum. Hum Genome Var. 2017 Oct 5;4:17040. doi: 10.1038/hgv.2017.40. PMID: 28983407; PMCID: PMC5628180.

Harripaul R, Noor A, Ayub M, Vincent JB. The Use of Next-Generation Sequencing for Research and Diagnostics for Intellectual Disability. Cold Spring Harb Perspect Med. 2017 Mar 1;7(3):a026864. doi: 10.1101/cshperspect.a026864. PMID: 28250017; PMCID: PMC5334248.

McKenna A, Hanna M, Banks E, et al. The Genome Analysis Toolkit: a MapReduce framework for analyzing next-generation DNA sequencing data. Genome Res. 2010;20:1297–303.

Murdock DR, Dai H, Burrage LC, Rosenfeld JA, Ketkar S, Müller MF, Yépez VA, Gagneur J, Liu P, Chen S, Jain M, Zapata G, Bacino CA, Chao HT, Moretti P, Craigen WJ, Hanchard NA; Undiagnosed Diseases Network, Lee B. Transcriptome-directed analysis for Mendelian disease diagnosis overcomes limitations of conventional genomic testing. J Clin Invest. 2021 Jan 4;131(1):e141500. doi: 10.1172/JCI141500.

Redon R, Carter NP. Comparative genomic hybridization: microarray design and data interpretation. Methods Mol Biol. 2009;529:37–49.

Retterer K, Scuffins J, Schmidt D, Lewis R, Pineda-Alvarez D, Stafford A, Schmidt L, Warren S, Gibellini F, Kondakova A, Blair A, Bale S, Matyakhina L, Meck J, Aradhya S, Haverfield E. Assessing copy number from exome sequencing and exome array CGH based on CNV spectrum in a large clinical cohort. Genet Med. 2015 Aug;17(8):623-9. doi: 10.1038/gim.2014.160. Epub 2014 Nov 6. PMID: 25356966.

Tarailo-Graovac M, Shyr C, Ross CJ, Horvath GA, Salvarinova R, Ye XC, Zhang LH, Bhavsar AP, Lee JJ, Drögemöller BI, Abdelsayed M, Alfadhel M, Armstrong L, Baumgartner MR, Burda P, Connolly MB, Cameron J, Demos M, Dewan T, Dionne J, Evans AM, Friedman JM, Garber I, Lewis S, Ling J, Mandal R, Mattman A, McKinnon M, Michoulas A, Metzger D,

Ogunbayo OA, Rakic B, Rozmus J, Ruben P, Sayson B, Santra S, Schultz KR, Selby K, Shekel P, Sirrs S, Skrypnyk C, Superti-Furga A, Turvey SE, Van Allen MI, Wishart D, Wu J, Wu J, Zafeiriou D, Kluijtmans L, Wevers RA, Eydoux P, Lehman AM, Vallance H, Stockler-Ipsiroglu S, Sinclair G, Wasserman WW, van Karnebeek CD. Exome Sequencing and the Management of Neurometabolic Disorders. N Engl J Med. 2016 Jun 9;374(23):2246-55. doi: 10.1056/NEJMoa1515792.

Wang K, Li M, Hakonarson H. ANNOVAR: functional annotation of genetic variants from high-throughput sequencing data. Nucleic Acids Res. 2010;38:e164.

## 6. Supplemental information

***Members of the Undiagnosed Diseases Network***

Maria T. Acosta, David R. Adams, Raquel L. Alvarez, Justin Alvey, Aimee Allworth, Ashley Andrews, Euan A. Ashley, Ben Afzali, Carlos A. Bacino, Guney Bademci, Ashok Balasubramanyam, Dustin Baldridge, Jim Bale, Michael Bamshad, Deborah Barbouth, Pinar Bayrak-Toydemir, Anita Beck, Alan H. Beggs, Edward Behrens, Gill Bejerano, Hugo J. Bellen, Jimmy Bennett, Jonathan A. Bernstein, Gerard T. Berry, Anna Bican, Stephanie Bivona, Elizabeth Blue, John Bohnsack, Devon Bonner, Lorenzo Botto, Lauren C. Briere, Gabrielle Brown Elizabeth A. Burke, Lindsay C. Burrage, Manish J. Butte, Peter Byers, William E. Byrd, John Carey, Olveen Carrasquillo, Thomas Cassini, Ta Chen Peter Chang, Sirisak Chanprasert, Hsiao-Tuan Chao, Ivan Chinn, Gary D. Clark, Terra R. Coakley, Laurel A. Cobban, Joy D. Cogan, Matthew Coggins, F. Sessions Cole, Heather A. Colley, Heidi Cope, Rosario Corona, William J. Craigen, Andrew B. Crouse, Michael Cunningham, Precilla D’Souza, Hongzheng Dai, Surendra Dasari, Joie Davis, Jyoti G. Dayal, Margaret Delgado, Esteban C. Dell'Angelica, Katrina Dipple, Daniel Doherty, Naghmeh Dorrani, Argenia L. Doss, Emilie D. Douine, Dawn Earl, David J. Eckstein, Lisa T. Emrick, Christine M. Eng, Marni Falk, Elizabeth L. Fieg, Paul G. Fisher, Brent

L. Fogel, Irman Forghani, Jiayu Fu, William A. Gahl, Ian Glass, Page C. Goddard, Rena A. Godfrey, Alana Grajewski, Andrea Gropman, Meghan C. Halley, Rizwan Hamid, Neal Hanchard, Kelly Hassey, Nichole Hayes, Frances High, Anne Hing, Fuki M. Hisama, Ingrid A. Holm, Jason Hom, Martha Horike-Pyne, Alden Huang, Yan Huang, Sarah Hutchison, Wendy Introne, Rosario Isasi, Kosuke Izumi, Gail P. Jarvik, Jeffrey Jarvik, Suman Jayadev, Orpa Jean-Marie, Vaidehi Jobanputra, Emerald Kaitryn, Shamika Ketkar, Dana Kiley, Gonench Kilich, Shilpa N. Kobren, Isaac S. Kohane, Jennefer N. Kohler, Susan Korrick, Deborah Krakow, Donna M. Krasnewich, Elijah Kravets, Seema R. Lalani, Byron Lam, Christina Lam, Brendan C. Lanpher, Ian R. Lanza, Kimberly LeBlanc, Brendan H. Lee, Roy Levitt, Richard A. Lewis, Pengfei Liu, Xue Zhong Liu, Nicola Longo, Sandra K. Loo, Joseph Loscalzo, Richard L. Maas, Ellen F. Macnamara, Calum A. MacRae, Valerie V. Maduro, AudreyStephannie Maghiro, Rachel Mahoney, May Christine V. Malicdan, Laura A. Mamounas, Teri A. Manolio, Rong Mao, Ronit Marom, Gabor Marth, Beth A. Martin, Martin G. Martin, Julian A. Martínez-Agosto, Shruti Marwaha, Jacob McCauley, Allyn McConkie-Rosell, Alexa T. McCray, Elisabeth McGee, Matthew Might, Danny Miller, Ghayda Mirzaa, Eva Morava, Paolo Moretti, Marie Morimoto, John J. Mulvihill, Mariko Nakano-Okuno, Stanley F. Nelson, Shirley Nieves-Rodriguez, Donna Novacic, Devin Oglesbee, James P. Orengo, Laura Pace, Stephen Pak, J. Carl Pallais, Jeanette C. Papp, Neil H. Parker, Leoyklang Petcharet, John A. Phillips III, Jennifer E. Posey, Lorraine Potocki, Barbara N. Pusey Swerdzewski, Aaron Quinlan, Deepak A. Rao, Anna Raper, Wendy Raskind, Genecee Renteria, Chloe M. Reuter, Lynette Rives, Amy K. Robertson, Lance H. Rodan, Jill A. Rosenfeld, Elizabeth Rosenthal, Francis Rossignol, Maura Ruzhnikov, Marla Sabaii, Ralph Sacco, Jacinda B. Sampson, Mario Saporta, Judy Schaechter, Timothy Schedl, Kelly Schoch, Daryl A. Scott, Elaine Seto, Prashant Sharma, Vandana Shashi, Emily Shelkowitz, Sam Sheppeard, Jimann Shin, Edwin K. Silverman, Janet S. Sinsheimer, Kathy Sisco, Edward C. Smith, Kevin S. Smith, Lilianna Solnica-Krezel, Ben Solomon, Rebecca C. Spillmann, Andrew Stergachis, Joan M. Stoler, Kathleen Sullivan, Jennifer A. Sullivan, Shirley Sutton, David A.

Sweetser, Virginia Sybert, Holly K. Tabor, Queenie K.-G. Tan, Amelia L. M. Tan, Arjun Tarakad, Herman Taylor, Mustafa Tekin, Fred Telischi, Willa Thorson, Cynthia J. Tifft, Camilo Toro, Alyssa A. Tran, Rachel A. Ungar, Tiina K. Urv, Adeline Vanderver, Matt Velinder, Dave Viskochil, Tiphanie P. Vogel, Colleen E. Wahl, Melissa Walker, Nicole M. Walley, Jennifer Wambach, Jijun Wan, Lee-kai Wang, Michael F. Wangler, Patricia A. Ward, Daniel Wegner, Monika Weisz Hubshman, Mark Wener, Tara Wenger, Monte Westerfield, Matthew T. Wheeler, Jordan Whitlock, Lynne A. Wolfe, Kim Worley, Shinya Yamamoto, Zhe Zhang, Stephan Zuchner
